# Elastic and Irreversible Bending of Tree and Shrub Branches Under Cantilever Loads

**DOI:** 10.3389/fpls.2019.00059

**Published:** 2019-02-05

**Authors:** Peter M. Ray, Marion Syndonia Bret-Harte

**Affiliations:** ^1^Department of Biological Sciences, Stanford University, Stanford, CA, United States; ^2^Institute of Arctic Biology, University of Alaska Fairbanks, Fairbanks, AK, United States

**Keywords:** biophysics, mechanics, viscoelasticity, retarded elasticity, irreversible strain, branch bending, cell-wall, instantaneous elasticity

## Abstract

Tree and shrub branches subjected to cantilever loads such as intercepted snowfall undergo, in addition to the familiar instantaneous elastic bending, a conspicuous retarded-elastic bending, which is commonly 30–50% of their instantaneous bending and occasionally even more. The resultant bending creep that occurs after loading also often includes a slow, time-dependent irreversible bending. These phenomena occur quite generally among woody plants of different major biomes, taxonomic groups, and structural types. We give some of branch bending viscoelasticity’s basic physical properties such as load dependence and stress relaxation. These properties belong to the secondary walls of branches’ xylem (wood) cells; some properties differ notably from those reported for primary cell walls, a difference for which we propose explanations. A method for separating the overlapping time courses of retarded-elastic and time-dependent irreversible bending shows that multiple retarded-elastic (“Kelvin”) elements of branches span a wide range of retardation times (a retardation spectrum, approximate examples of which we calculate), and that irreversible bending can occur in different cases either only in the first few h after loading, or more extensively through 24 h, or (rarely) for several days. A separate time-independent irreversible bending, permanent set, involving a substantial yield stress, also occurs. In three species of shrubs rapid irreversible bending began only several (up to 24) h after loading, implying an unusual kind of viscoelasticity. Deductions from the dynamics of bending suggest that retarded elasticity can help protect branches against breakage by wind gusts during storms. Irreversible bending probably contributes both to the form that tree and shrub crowns develop over the long term, involving progressive increase in the downward curvature and/or inclination of branches, and also to certain other, more specialized, developmental changes.

## Introduction

The elasticity of woody-plant branches toward bending under cantilever loads seems obvious, and a number of papers have used bending moduli, measured or inferred for branches, to analyze or predict branches’ behavior under loadings such as by snow (*e.g.*, Schmidt and Pomeroy, 1990), ice, wind (*e.g.*, Sellier and Fourcaud, 2009), or further shoot growth (*e.g.*, Alméras et al., 2002). However, the viscoelastic (time-dependent) aspects of branch bending seem, from the botanical literature, to be largely unappreciated. These aspects include a rather conspicuous retarded-elastic component, often accompanied by a certain amount of irreversible bending. Although the botanical literature contains elegant accounts both of plant axial bending (Niklas and Spatz, 2012, pp. 161–174) and of viscoelasticity (Niklas, 1992, pp. 93–102; Niklas and Spatz, 2012, pp. 134–144), the only plant material that these accounts mention as showing viscoelasticity is the thin primary walls of growing cells. We have found almost no descriptions of woody branch viscoelasticity in the botanical literature, or elsewhere.

The occurrence, in wood, of retarded elasticity was recognized long ago (Kitazawa, 1947; Grossman, 1954; Kollmann, 1961; Kollmann and Coté, 1968). This is relevant to woody branch bending because these structures typically owe their mechanical support mainly to their xylem’s (wood’s) thick secondary cell walls. Although some contemporary expositions of wood’s mechanical properties do not even mention the time-dependent component of its elasticity (*e.g*., Mattheck and Kubler, 1995; Bowyer et al., 2007), it has been studied rather extensively (*e.g*., Schniewind, 1979; Dinwoodie, 2000; Ouis, 2002, and refs. there cited), and is recognized as “mechanical damping” in the literature on swaying of tree trunks in winds (*e.g.*, Sellier and Fourcaud, 2009). In order specifically to characterize the mechanical basis of branch bending, Hogan and Niklas (2004) made bending measurements on strips of linden (*Tilia americana*) wood. These nicely demonstrated this wood’s viscoelasticity, including both retarded-elastic and irreversible bending, but did not include other features, nor the species breadth, upon which we report here. Several more recent studies model wood cells’ viscoelastic behavior (Engelund and Svensson, 2011; Huc and Svensson, 2018, and refs. there cited).

We first encountered viscoelastic branch bending while investigating the bending responses of arctic tundra shrubs to the imposition and removal of snow loads. In testing, for comparison, woody species from other latitudes and biomes, we found that the kinds of viscoelastic behavior found in arctic shrubs mostly occur quite generally in branches from all biomes. This paper describes these kinds of behavior (plus a few notable exceptions that we discovered), illustrated by data from a number of species from different biomes. The concepts that embrace this information are needed for analyzing fully the behavior of tree and shrub branches and trunks in response to the kinds of loading they are subject to, as well as for explaining certain developmental aspects of the over-all form of many tree and shrub crowns.

**FIGURE 1:**
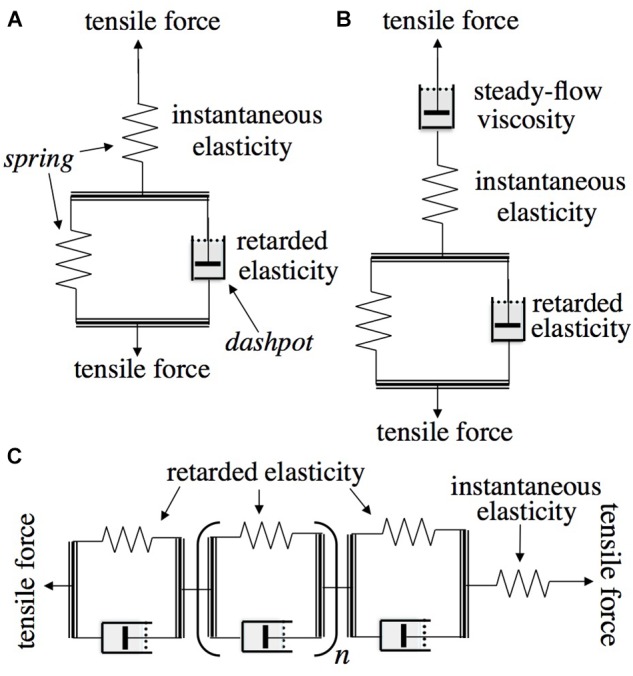
Rheologically conventional mechanical models representing quantitative features of viscoelasticity. Arrows show the directions in which tensile or compressive stress is applied. Double lines represent rigid yokes that couple two mechanical components together in parallel. **(A)** “Standard solid” model consisting of a Kelvin element (below: retarded elasticity), in series with an unretarded spring (above: instantaneous elasticity). **(B)** The same in series with an unrestricted dashpot (“steady-flow viscosity”) enabling the material to also deform irreversibly (“Burgers model”). **(C)** “Generalized standard solid” model containing, in series, multiple Kelvin elements with different viscosities and/or compliances.

As background and for use in what follows, Figure 1 depicts the type of mechanical models employed in rheological theory (Findley et al., l976/1989) to represent, by analogy, viscoelastic behavior and to derive its quantitative characteristics. When a pulling (tensile) or a pushing (compressive) load is imposed upon the model in Figure 1A, which is called the “standard solid” (Findley et al., l976/1989) or “general linear substance” (Jaeger, 2012), it will undergo an immediate elastic extension or contraction (respectively) of the upper, unrestricted spring (instantaneous elastic strain), followed by a gradual, time-dependent further movement (“creep”) as the dashpot (piston in viscous fluid-containing cylinder), under the load that initially acts on it, extends or contracts, allowing the spring that is in parallel with it to do the same. This gradually shifts the imposed load from the dashpot onto that spring until this latter comes to bear the entire load, ending further displacement. If the model is then relieved of its load, the unrestricted spring will immediately return to its original length (instantaneous elastic recovery), but the second spring (restricted by the dashpot) can do so only by the load that it still bears, time-dependently driving a reverse displacement of the dashpot. This latter recovery shows that the previous post-loading creep was reversible, and is hence termed a “retarded elastic” strain. This kind of behavior is characteristic of woody branch bending, as shown below. The parallel combination of a spring and a dashpot in Figure 1A is termed a “Kelvin” or “Voigt” element.

The occurrence of irreversible bending during branch cantilever loading can be included in the rheological model by inserting in series, as at the top in Figure 1B, a second, “steady-flow” dashpot. This, being unrestricted by any in-parallel spring, will extend or contract whenever a load is imposed, adding an irreversible (unrecoverable) strain to the elastic (recoverable) strains developed in the other elements of the system under a load. This is called a “Burgers” model (Findley et al., l976/1989). The modeled elements just noted reflect molecular mechanisms for supporting (or resisting) imposed forces and for recovering after being relieved from them.

Besides compression and extension of xylem cell wall material on the lower and upper sides, respectively, of a branch subjected to a cantilever load, bending could also involve changes in the shape of wood cells or even some degree of collapse or of separation of cells on the respective sides, or also flow, through the stem cross section from one side of the stem toward the other, of water that is located within the cell walls or lumens (cell chambers) (“poroelasticity,” *cf.* Hu and Suo, 2012). The total of these effects, to whatever extent they occur and resist or allow bending, are collectively represented by the conventional viscoelastic parameters reviewed above. One other, the St-Venant element, will be introduced in the Discussion.

Although in work with artificial materials such as synthetic polymers the various kinds of viscoelastic behavior are regarded as purely mechanical (physical) processes, with unchanging parameters, the objects tested here are living, biological specimens whose behavior can be subject to biologically caused variations. While the present measurements deal with physical behavior, certain observed changes that we encountered in branch viscoelasticity that may be of biological origin will be noted later; variability and reproducibility of the ordinary elastic behavior of these specimens may also, unknowingly, be affected by biological variations.

The goal of this study is to quantitatively evaluate the viscoelastic behavior of branch bending under cantilever loads, across a range of woody species. Although, depending on the branch, either a standard solid or a Burgers model qualitatively imitates the branch bending studied here, it turns out that quantitatively neither model agrees at all accurately with branch bending. We show below that a considerably more complicated model, involving a spectrum of Kelvin elements with different time constants, plus at least two kinds of irreversible bending, is needed to account for branch bending. The biological significance of these components is considered in the Discussion.

## Materials and Methods

### Plant Material

Stem segments usually between 25 and 30 cm long and 6–9 mm in diameter were harvested from shrubs and from the lateral branches of trees, located either in natural vegetation in the states of Alaska, Washington, California, Arizona or New Mexico, or (for natives of other states or countries) in greenhouses or in outdoor horticultural plantings in one of the mentioned states. After removing any leaves, the segments were kept moist within plastic bags at 3–5°C until used. Species identities were ascertained from general botanical knowledge of the authors and/or by reference to local floras (Hultén, 1968; Hitchcock and Cronquist, 1973; Carter, 1997; Cody, 2000; Karlsson, 2000; Baldwin et al., 2012) and, for greenhouse and horticultural plantings, to manuals of cultivated plants (Bailey, 1949; McMinn and Maino, 1956; Rehder, 1986/2001; Sibley, 2009). Generally at least 4 branches from any given species, usually from different individuals, were tested in the standard bending assay. Supplementary Table S1 gives source and native habitat information for species for which individual bending data are given here.

### Bending Assay

**FIGURE 2:**
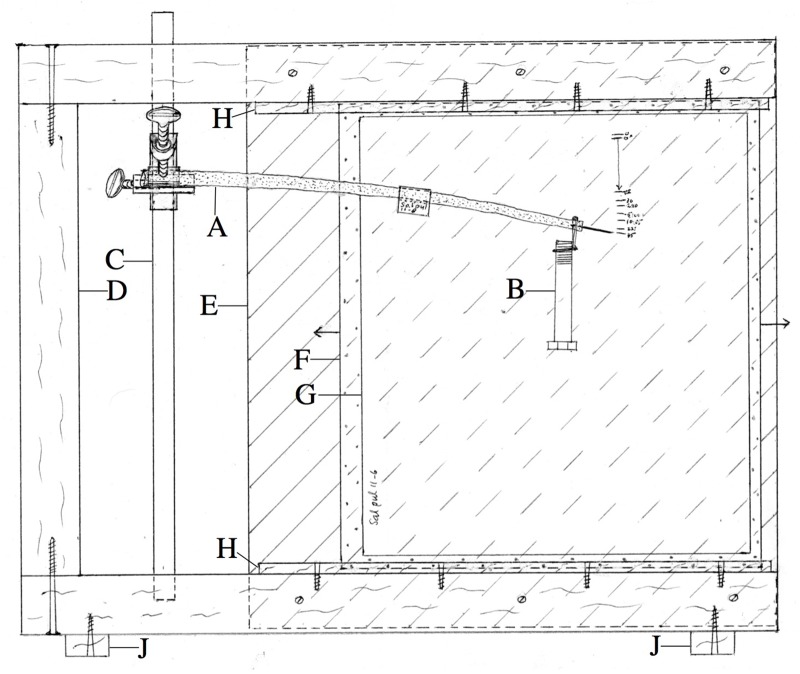
Apparatus used for bending experiments. **(A)** Branch segment being measured, with apically inserted pin whose elevation is recorded on record paper G immediately behind the specimen. **(B)** Weight (load). **(C)** Rod to which branch segment’s basal end, protected by a hard, semicylindrical polyethylene sleeve, is clamped by the bar-to-bar clamp. **(D)** Frame made from 2 × 4” timber. **(E)** Rear plywood panel, in front of which (behind branch segment) rides plexiglas panel **(F)**, to which record paper **(G)**’s corners are attached by “Scotch” tape. Wood strips **(H)** hold **(F)** vertical, but allow it to be slid laterally (arrows) for non-overlap of records from successive parts of experiment. **(J)** Stabilizing feet project beyond sides of frame. Width of apparatus, 45 cm.

The basal end of a stem segment 25–35 cm long and usually between 6 and 10 mm in diameter was clamped firmly in an apparatus (Figure 2) after a pin about 1.5 cm long had been inserted into the xylem at the segment’s apical end to act as a position pointer. On record paper mounted immediately behind the stem (Figure 2G) we marked the pointer pin tip’s elevation initially, and after a load initially giving usually 3–4 cm of downward deflection was hung momentarily from the segment’s apical end and immediately removed (giving a “permanent set” deflection), and then over time when, and after, the same load was re-applied and left in place, and when and after it was later removed. All bending experiments described here were performed at about 21°C, except where noted otherwise.

Each stem segment’s deflections were later measured, on the record, to the nearest 0.5 mm and entered into a spreadsheet from which time course plots could be obtained.

The fresh weight of each branch segment was determined before and after the bending test, and later its dry weight by drying to constant weight at 60°C, to verify that, despite some evaporative water loss during the bending tests, the segment’s water content remained >30% of its dry weight, a range over which branches’ viscoelastic properties are essentially independent of water content (Dinwoodie, 2000, pp. 54–55). Its length and its apical, midpoint and basal diameters were also measured.

Additional, significant details of our bending measurement technique are given in Supplementary Material.

### Stress Relaxation

A stem segment long enough that its tip would extend just beyond the bending apparatus’ frame (Figure 2D) and rear panel (E) was mounted in the apparatus, with record paper positioned immediately behind the stem’s pointer tip by sliding the paper-bearing plexiglas panel (F) outward (to the right in Figure 2). The segment’s tip was connected vertically to a spring balance sensitive to ±2 g (Homs Model 2 Laboratory Scale, 1000 × 10 g scale, Douglas Homs Corp., Belmont, CA, United States) which was held directly above the stem tip by a 3-prong clamp that was attached to the portion of the clamping rod (C) that extended above the top of the apparatus’ frame. By raising the spring balance at zero time, the stem tip was deflected upward and its elevation was marked immediately on the record paper, and the spring balance’s load was recorded. The distance of the spring balance above the branch tip was adjusted, over time, to maintain the tip at its initial post-deflection elevation, while the load registered by the spring balance was recorded at intervals between the mentioned adjustments.

### Load Dependence

This was determined by applying successively increasing loads to a given specimen. For each load the sequence of steps described above under “Bending assay” was followed, except that the branch was unloaded 30 min after post-loading creep had begun, and post-unloading recovery was followed also for just 30 min, after which the next higher load was applied and the same sequence of steps was followed.

After the final 30 min recovery period, the branch was loaded with a moderate weight (substantially below the maximum that had previously been applied) which remained in place for 12 h or more, to attain nearly complete response of most of the specimen’s Kelvin elements. It was then unloaded and followed for another 12 h or more until its retarded recovery was complete. This gave the ratio (*r*) between its retarded recovery after, and before, the first 30 min after unloading. Based on the geometry of exponential progress curves for alternating 30 min periods of load and no load, as compared with complete loading and unloading equilibration, the retarded recovery during each of the 30 min unloading periods after different loads was multiplied by the factor (1 + 2*r*) to give approximately what the full retarded-elastic recovery deflection for that load would have been if that loading had lasted long enough for complete equilibration with the load and if the subsequent post-unloading recovery had gone to completion. The factor (1 + 2*r*) holds if the complete retarded-recovery’s first *t*_1/2_ was not longer than the loading and unloading interval (30 min in this case), as was true for the branch segments tested in this work.

The increments in permanent set and time-dependent irreversible bending during the 30 min loadings were very small (compared to the instantaneous, creep, and recovery deflections). The most useful display of these irreversible bending data was to plot the cumulative deflection, *i.e.*, the sum of all the individual irreversible increments, from each successive load, from the first-imposed load up to and including that for the given data point.

### Retarded Recovery Curve-Fitting

The curve-fitting program of KaleidaGraph^1^ was used, employing either of two methods. The first method started with the sum of 6 terms, each like equa. (1), with initially pre-chosen *b*_i_ values spread evenly over the time range of the recovery time course data, for which terms the computer calculated the set of *a*_i_ values that gave the best fit to the data points that could be obtained from those *b*_i_ values, and then repeated this calculation iteratively after the operator adjusted some of the *b*_i_ values (and/or added an additional exponential term) on a trial-and-error basis until the best possible fit, usually a near-perfect or perfect fit, was obtained. A “perfect fit” means a calculated curve that passes directly through, or at least touches, all of the recovery time course data points.

In the second method the computer calculated both *a*_i_ and *b*_i_ values for 4 terms like equation (1), which it summed as in equation (2) (the curve-fitting program could handle at most only 4 bivariate terms). This method gave less perfect fits, but is mentioned because all its coefficients’ values were obtained without any operator direction. Its results agreed qualitatively with the Method 1 results in that its terms were spread, by the computer’s choice, over the several log decades of the recovery curve data, as deduced from data in Table 1, below. Method 2’s less perfect agreement with the data points indicated that more than 4 terms were needed to obtain a perfect fit.

**Table 1:** Time course parameters for post-unloading retarded elastic recovery of cantilever-bent stems.

Species^1^	*t*_0.1_	*t*_1/4_	1st t_1/2_	2nd t_1/2_	3rd t_1/2_	4th *t*_1/2_^4^
			*min*^2^			
*Adenostoma fasciculatum*	0.61 ± 0.17	4.8 ± 1.6	64 ± 28	285 ± 5	388 ± 102	675 ± 135
*Arctostaphylos glandulosa*	0.83 ± 0.38	6.6 ± 6.6	75 ± 15	417 ± 126	820 ± 11	754^7^
*Diospyros virginiana*	0.33 ± 0.01	1.8 ± 0.2	27 ± 8	208 ± 53	365 ± 15	400 ± 75
*Genista monspessulana*	0.72 ± 0.22	12.1 ± 0.9	123 ± 28	300 ± 50	423 ± 8	422 ± 57
*Liriodendron tulipifera*	0.20 ± 0.02	0.44 ± 0.01	3.9 ± 0.6	31 ± 3	149 ± 14	208 ± 19
*Metasequoia glyptostroboides*^3^	0.24 ± 0.12	1.0 ± 0.6	55 ± 36	388 ± 352	532 ± 439	92^7,8^
*Morus alba*	0.23 ± 0.05	1.2 ± 0.1	8.8 ± 3.2	65 ± 23	143 ± 21	187 ± 13
*Populus tremuloides*	0.26 ± 0.05	1.2 ± 0.2	15 ± 1	284 ± 99	700 ± 160	520^7^
*Pseudotsuga menziesii*^3^	0.27 ± 0.12	1.0 ± 0.35	13 ± 7	82 ± 40	214 ± 92	253 ± 58
*Salix glauca*	0.28 ± 0.04	1.1 ± 0.2	11 ± 2	79 ± 4	177 ± 15	170^7^
*Umbellularia californica*	0.36 ± 0.11	1.0 ± 0.3	23 ± 14	245 ± 165	425 ± 175	300^7^
Mean of (*t_x_*/1st *t*_1/2_) ± SE^5^	0.022 ± 0.0034	0.092 ± 0.0085	1.0 ± 0	8.2 ± 1.2	19.9 ± 3.7	21.8 ± 3.9
Ratio for exponential curve^6^	0.301	0.584	1	1	1	1


*Numbers are the intervals (in min) between the time of unloading and either the one-tenth time (*t*_*0.1*_), the quarter-time (*t*_*1/4*_), or the first half-time (1st *t*_*1/2*_) or, to the right of this, the intervals between subsequent half-times (time intervals for the branch tip to progress half way, toward its final elevation, from its elevation at the preceding halfway point, *cf*. Figure 6).*

*^*1*^Sources (provenances) of the branches used in this experiment are given in Supplementary Table S1.*

*^*2*^Means for recovery time courses for two different branches of each species, ±average deviation from this mean, except for numbers lacking a±, for which see footnote 4.*

*^*3*^These species are softwoods (conifers); all the rest are hardwoods (flowering plants).*

*^*4*^In 5 of the species (lacking a±) one of the two recovery time courses dropped so close to 0 by the time of a 4th *t*_*1/2*_ interval that this interval could not be reliably estimated. The similarity, between most of the 4th *t*_*1/2*_ intervals that were estimated, and the preceding 3rd *t*_*1/2*_ interval, would be expected for the final, exponential tail of a multi-exponential-term retardation spectrum.*

*^*5*^Mean of the ratios of each of the numbers above, to the same row’s 1st *t*_*1/2*_ value, ± SE for this set of ratios, *n* = 22, except 17 for the 4th *t*_*1/2*_.*

*^*6*^Ratio of the given interval to the 1st *t*_*1/2*_, for a simple exponential time course.*

*^*7*^A 4th *t*_*1/2*_ interval was estimated for one out of the two recovery time courses, for the reason given in footnote 4, so there is no associated ± value.*

*^*8*^The 3rd *t*_*1/2*_ interval for this recovery was also 92 (the two recoveries differed greatly, quantitatively, from one another).*

Additional information about details of our curve-fitting are given in Supplementary Material, and an example of retarded-recovery curve-fitting by each of the above methods is shown in its Supplementary Figure S1.

### Retardation Spectra

Recovery values [ε(*t*)s] for any given branch’s post-unloading retarded-recovery time course’s best curve-fit equation (most perfect fit that we obtained, to the recovery data), were, without that equation’s *CL* factors, entered into the computer using a time scale of 2.154-fold intervals (=10^1/3^) extending over the ca. 4-decade time range of the data. Each such set was differentiated by the computer with respect to log_10_*t* over each of the mentioned intervals to obtain, following equation (4), an estimate of fractional compliance per unit of log time [ρ(τ)] for each interval. The computer then drew a smooth curve, of its choice, through a plot of these calculated points.

## Results

**FIGURE 3:**
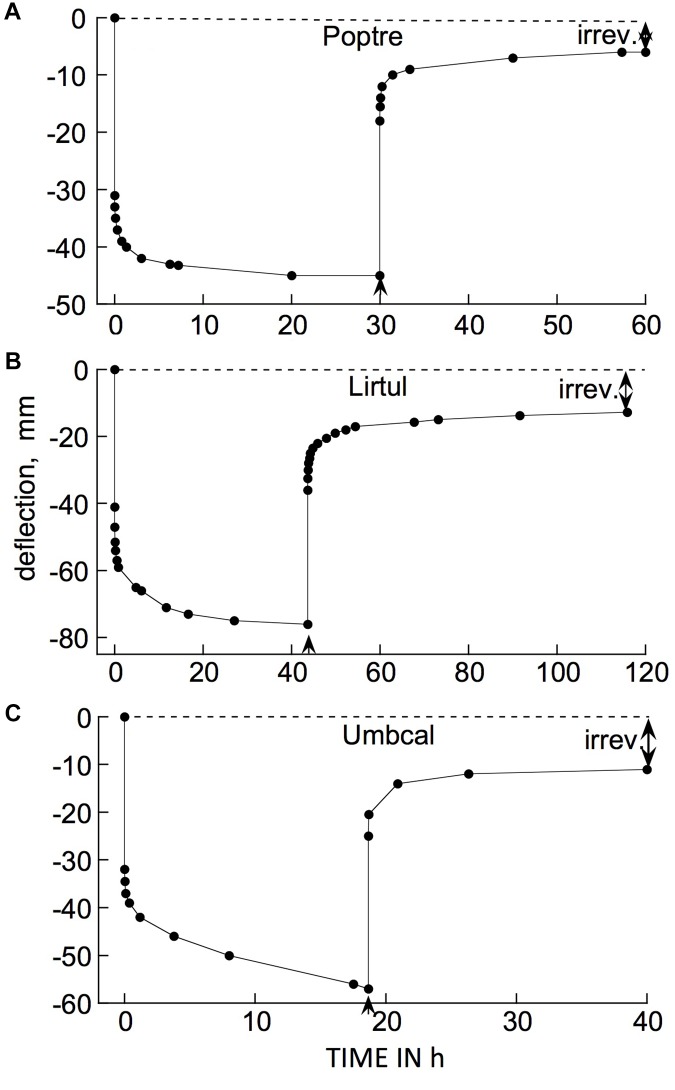
Time courses of cantilever load-induced bending of branch segments, ca. 30 cm long, from three tree species. **(A)** *Populus tremuloides* (“Poptre”); **(B)** *Liriodendron tulipifera* (“Lirtul”); **(C)** *Umbellularia californica* (“Umbcal”). Loads were applied at zero time, and removed at ↑. The first data point below 0 deflection, at zero time, shows the “instantaneous” (≤3 s) deflection. Subsequent marker points show subsequently occurring creep (post loading), and instantaneous and retarded recovery post unloading. The early, rapid, post- loading and -unloading parts of these curves are very steep because of the long time scale needed to show the later, much slower parts of the creep and recovery responses. “Irrev.” denotes irreversible bending (double-headed arrows) that had occurred during the period under load.

Examples of typical responses, to a cantilever load, of branch segments from several woody species at 21°C are shown in Figure 3. The bending response to a load and to its subsequent removal consists of the following components:

(a) A usually small, and often negligible, “permanent set” or irreversible bending upon initially applying the load and immediately removing it. For most of the branches in the data included here, at the loads we normally employed, permanent set was too small to appear in the plots presented. It became significant at higher loads (see later).
(b) An immediate (“instantaneous”) bending when the load was reapplied and left in place.
(c) A time-dependent additional bending (“creep”) of gradually declining rate that in most cases reached completion within about 24 h, but sometimes only declined by that time to a low but non-zero rate.
(d) When the load was then removed, an immediate, partial recovery from bending that was generally equal, or close, to (b), indicating that (b) and (d) represent instantaneously reversible*, i.e.*, “instantaneous” elastic, bending.
(e) A time-dependent, post-unloading further recovery toward the branch tip’s initial elevation, which ceased usually within about 24 h after unloading. This recovery was qualitatively, and in many cases quantitatively, a mirror image of the time-dependent post-loading creep (c), indicating that time-dependent recovery, and presumably a corresponding part of (c), are due to retarded elasticity. The recovery occurring during this phase of the experiment is sometimes called “creep recovery” or “strain relaxation.”
(f) A usually small, and sometimes negligible, deflection, below the branch tip’s initial (post permanent set) elevation remained after post-unloading recovery ceased. This remainder represents an irreversible deformation that occurred during the period subsequent to loading. This will hereafter be called time-dependent irreversible bending.

Whenever significant time-dependent irreversible bending was found at the end of a loading/unloading test, the creep component (c) under load was larger than the retarded elastic recovery (e) after unloading, by about this same amount. This suggests that when significant post-loading irreversible bending is detected after a load has been imposed and then unloaded, this irreversible strain developed gradually, along with the development of retarded-elastic bending during phase (c), but did not increase after unloading (phases d and e). This agrees with rheological theory which holds that irreversible strains develop only while external forces are being imposed (Findley et al., l976/1989). Thus, when irreversible bending occurs during post-loading creep (c), this creep is evidently a composite of retarded-elastic plus time-dependent irreversible bending (both of these falling within the general term viscoelasticity).

**FIGURE 4:**
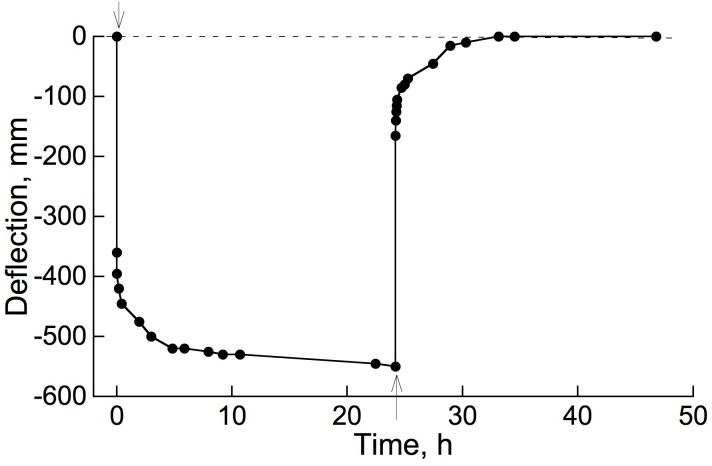
Bending of an attached branch of the shrub *Salix glauca*, outdoors, when a 266 g weight was hung from the branch 125 cm from its basal end. Distance of the weight’s attachment point above ground was measured repeatedly to record the response to loading and unloading.

The occurrence of retarded, in addition to instantaneous, elastic bending of intact, attached branches of trees or shrubs out of doors can easily be demonstrated by hanging a suitable weight onto a distal part of an inclined or horizontal branch system and measuring, over time, the weight’s elevation above the ground surface (Figure 4). The extent of deflection here was much greater than in Figure 3 (even though loads were similar), because here an entire branch system was bending, rather than the relatively short, isolated stem segments used in our bending apparatus (Figure 2). Minor air motions that occur frequently outdoors significantly perturb the elevation of an entire branch, probably explaining Figure 4 curve’s departure from the simple concave form in Figure 3.

The time courses in Figure 3 exemplify a general phenomenon in woody branches. We have obtained bending records comparable to Figure 3 from many other woody species from all the major natural biomes. Branches from shrubs and trees gave mean retarded-elastic relative to instantaneous compliances that were not significantly different. However, the ratio of mean retarded-elastic to instantaneous elastic compliance (expressed as a%) was significantly larger in hardwoods (flowering plants or Angiosperms) (47.3% ± 1.14 (SE), *n* = 168 species) than in softwoods (Gymnosperms, mostly conifers) (34.4% ± 1.74, *n* = 31 species) (1-way ANOVA, *F*_1,197_ = 21.7202, *P* = < 0.0001). For irreversible bending rate, on the other hand, because of much greater variation in the data a difference between hardwoods and softwoods was only marginally significant (mean for hardwoods 2.30 ± 0.286 % per h and for softwoods 1.13 ± 0.083; 1-way ANOVA, *F*_1,197_ = 3.0343, *P* = 0.0831).

Although the bark of woody branches almost invariably contains mechanical tissues (phloem fibers; cork), the bending of temperate and boreal tree and shrub branches more than about 6 mm in diameter (as tested here) seems to be governed mainly by the elastic properties of their xylem. In a few cases we were able to strip off the bark from such a branch and found that both instantaneous and retarded components of its elastic compliance increased only modestly. With one branch of the willow *Salix pulchra*, for example, bark removal increased the instantaneous compliance (ratio of bending deflection to load) by 17% and the retarded compliance by 25%, whereas stripping another branch of this species increased both of these compliances by only 7%. Removing the stem’s most peripheral mechanical layer would be expected to increase the stem’s bending compliance, since this layer undergoes the greatest strain (extension or compression) of any of the stem’s tissue layers during bending and therefore tends to bear the largest tensile or compressive stress (on the stem’s upper or lower sides, respectively). However, that these stems’ bending behavior was basically the same before and after removing their bark implicates their xylem as principally responsible for their elastic behavior.

### Stress Relaxation

Materials that have significant retarded as well as instantaneous elasticity, as in Figure 1A, can undergo *stress relaxation*, a time-dependent decline in stress after the object has been deformed (strained) to a certain extent and is then held at that level of strain while measuring the load that holds it at that strain (Findley et al., l976/1989). Study of Figure 1A will show that the standard solid model should undergo stress relaxation under these circumstances.

**FIGURE 5:**
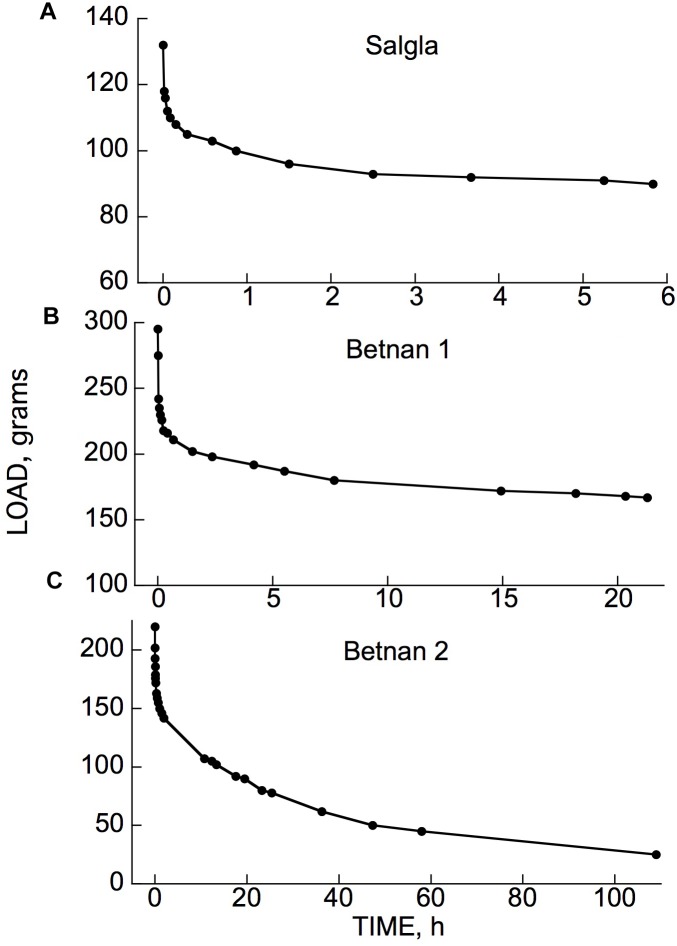
Stress relaxation in cantilever-loaded branch segments from **(A)** *Salix glauca* (“Salgla”) and **(B,C)** *Betula nana* (“Betnan”). After initial load was applied (first marker point), the branches were kept at the thereby-imposed deflection of about 4 cm while the load that maintained this deflection was recorded. A repeat run using the branch segment in **(B)** gave an almost identical time course. **(C)** Is for a different branch than that in **(B)**. Note the offscale ordinate zero-load points for the plots **(A,B)**, showing that (unlike **C**) the load remained far above zero at the end of the experiment, when virtually no further relaxation was occurring.

Figure 5 gives data showing that under a constant bending strain, stress relaxation occurred in branches of two woody species which, like all those we have tested, possess instantaneous plus retarded elasticity (as in Figure 1A).

As will be evident from Figure 1B, if a material can develop irreversible strain under an imposed stress, this straining capacity would also contribute to stress relaxation (*e.g.*, Cosgrove, 1997). If the material possesses unrestricted (“steady-flow”) viscosity, as the Burgers model (Figure 1B) does (its upper dashpot, which is not restricted by any in-parallel spring), stress at constant strain should eventually relax down to zero. However, the plots in Figure 5A,B suggest that the load on these branches was relaxing only down to a level moderately below the initially imposed load, not to zero. This suggests that these branches did not possess a steady-flow bending viscosity, or if they do, that this viscosity increased, with time and/or strain, toward infinity. However, with another branch (Figure 5C) of the same species as in Figure 5B (*Betula nana*), a much larger relaxation occurred. The load seems to have been declining toward 0, although it had not completely reached 0 after 4.5 days of relaxation. This branch apparently behaved, at least qualitatively, like a Burgers model (Figure 1B).

To explore this further, the time course of time-dependent irreversible bending must be obtained, to do which the time course of retarded-elastic bending must first be considered.

### Time Characteristics of Retarded-Elastic Bending

Because of the abovementioned overlap, during bending creep, between retarded-elastic and irreversible bending, the time courses of these respective deflections must be separated to obtain either one. The time course of a branch’s retarded-elastic deflection should be obtainable from the time course of post-unloading recovery, because in that part of the experiment no external force is acting to cause irreversible deformation. A basic point from the Boltzmann superposition principle of rheology (Findley et al., l976/1989), and from the mechanical models in Figure 1, is that the time course of a retarded recovery should be the exact reverse of the time course by which the given retarded-elastic strain was generated.

**FIGURE 6:**
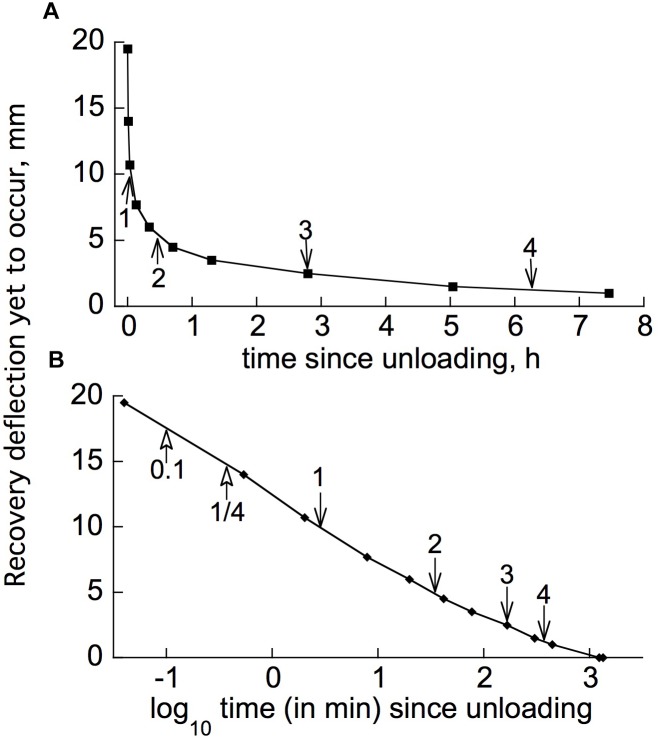
Post-unloading, retarded recovery of a branch segment from *Liriodendron tulipifera*. **(A)** Linear plot of the earlier part of the time course (the first ∼1/3 of the time to complete recovery, comprising ∼95% of the total recovery deflection), successive half-times [*t*_1/2_(1), *t*_1/2_(2), etc.] being marked by arrows. Difference between the tip’s elevation at various times after unloading, and its final elevation when recovery ceased about 26 h later, is plotted (“deflection yet to occur”). **(B)** Log_10_ time plot of the same data, with arrows marking the *t*_1/2_s, plus *t*_0.1_, and *t*_1/4_ (times for 1/10 or 1/4, respectively, of the complete response to occur).

Figure 6A gives, on an expanded time scale, the earlier part of a retarded-elastic recovery, plotted as the difference between the branch tip’s elevation at any given time (from immediately after unloading) and its final elevation ca. 24 h after unloading. Now the time course of retarded-elastic bending, or of retarded post-unloading recovery, for the “standard solid” model of retarded elasticity (Figure 1A) should be a simple, exponential approach (equation 1, below) to the finally attained elevation (Findley et al., l976/1989). Although the progress curve in Figure 6A may superficially look exponential, it is actually far from it. An exponential curve approaches its end point with a constant half-time (*t*_1/2_), *i.e.*, a constant time interval to progress from any given point on the curve to the point that is half as far away from the variable’s final value. Arrows placed at successive *t*_1/2_ points along the curve in Figure 6A show that the data conflict markedly with this. Values of *t*_1/2_ intervals beyond the first one increase progressively and substantially.

**FIGURE 7:**
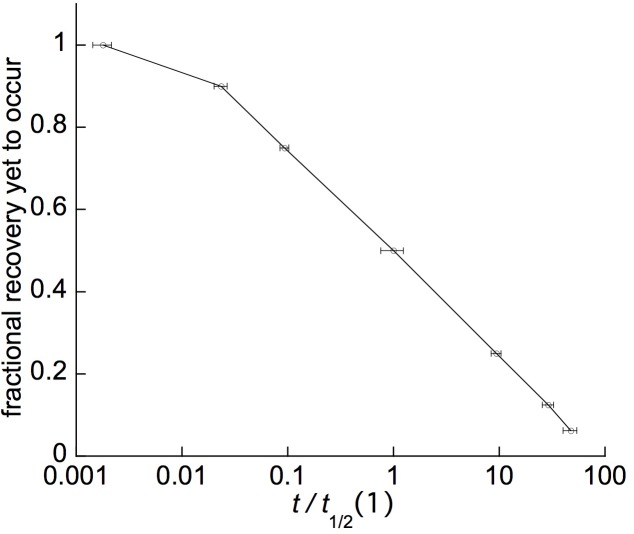
Average retarded-elastic recovery curve computed from the data in Table 1. Marker points show the means and SEs (error bars, *n* = 22, except 17 for 4th *t*_1/2_) for the fractional time (FTV) values (*t*_0.1_, *t*_1/4_, 1st*t*_1/2_, 2nd*t*_1/2_, etc.) used in Table 1, each of which was normalized (giving relative [RFTV] values) by dividing by its curve’s 1st *t*_1/2_, putting the 1st *t*_1/2_ at the 1 point of the abscissa’s logarithmic relative-time scale. The values used for successive time points beyond the 1st *t*_1/2_ are the total time up to that point, i.e., the 1st *t*_1/2_ for the given curve, plus the successive *t*_1/2_ intervals shown for it in Table 1 up to that for the *t*_1/2_ in question. The error bar shown for the 1st *t*_1/2_ is its SE, relative to the mean 1st *t*_1/2_, for this FTV in the raw data, before normalization of the data by dividing by 1st *t*_1/2_ values (the normalized 1st *t*_1/2_ of course has a zero SE).

Plots of these retarded-elastic recovery data against the log of time (*e.g.*, Figure 6B) generally fell close to, and sometimes almost exactly upon, a straight line. This has been found previously for the creep recovery (“strain relaxation”) of various artificial viscoelastic materials (Krauss and Eyring, 1975, pp. 208–223) and for related behavior of various biological objects. Data obtained from log-time plots of duplicate recovery tests on 11 woody species are given in Table 1. The mean time course (Figure 7) was obtained by averaging fractional recovery times (Table 1’s columns), for all these recoveries, after normalizing the data by dividing each fractional time (in min) by that run’s first *t*_1/2_ to correct for different time courses’ midpoints being positioned differently on the log time scale (this normalization yielded, for most of the fractional time means, about half the variance of the raw data). This plot (Figure 7), not surprisingly, is almost exactly log-linear over most of the range of its data. It, and the successive *t*_1/2_ intervals given in Table 1, show that the progressive increase in *t*_1/2_ values over the course of branch retarded-elastic recovery seen in Figure 6A is quite general among the tested species, although the actual *t*_1/2_ values varied rather widely among them (Table 1).

These findings imply that branches’ retarded-elastic behavior is due to multiple viscoelastic elements having *t*_1/2_s that are spread over much of the recovery time course beyond its first *t*_1/2_. This spread, furthermore, extends down through most or all of the early part of the recovery curve preceding its first t_1/2_. This is shown by the times required for these curves to progress 1/10 of (*t*_0.1_), or 1/4 of (*t*_1/4_), the way to completion (Figure 7 and Table 1) being much shorter than for a simple, exponential response (single Kelvin element). For the latter, from equa. (1) below, *t*_0.1_ = 0.301^∗^*t*_1/2_, and *t*_1/4_ = 0.584^∗^*t*_1/2_. The mean *t*_0.1_/*t*_1/2_ and *t*_1/4_/*t*_1/2_ ratios given in the next to last row of Table 1 and plotted in Figure 7 are much less than these numbers, and significantly so since their SEs are much smaller than their differences from the respective exponential ratios. These large departures from the early-time form of a simple exponential curve imply that the retarded elastic response includes Kelvin elements with *t*_1/2_s far below the response’s first over-all *t*_1/2_, *i.e.*, close to, and/or possibly even into, the curve’s zero-time or what in the present type of assay is recorded as the “instantaneous,” elastic response. Similar retarded-elastic behavior occurs in many synthetic polymeric materials (Ferry, 1980).

### Curve-Fitting Analysis of Retarded-Elastic Recoveries

The rheological model (Figure 1C) that can duplicate the foregoing kind of progress curve, called a “generalized Kelvin Model” (Findley et al., l976/1989), consists of multiple Kelvin elements, in series, having a wide range of “retardation times” (τ), the temporal parameter generally employed in the mathematical theory of rheology (τ = 1.443^∗^*t*_1/2_; the *t*_1/2_ is simpler for visually evaluating progress curves like Figure 6 so is mainly used here). This model includes (like Figure 1A) a single un-retarded spring to provide for instantaneous elasticity. If, to allow for irreversible bending, an unrestricted dashpot in series with the other elements were also included, as at the top of Figure 1B, the model would be called a generalized Burgers model.

We confirmed that the model in Figure 1C applies to woody branch retarded-elastic bending by matching, by computer curve-fitting, their recovery time courses to the equation that applies to Figure 1C. The retarded-elastic recovery of a single Kelvin element that has come into elastic equilibrium with a certain load *L* and is then unloaded, has the simple exponential form:

(1) $$\varepsilon (t)=CL*e^{-bt}$$

where ε(*t*) is the bending strain (away from the final elevation) that remains at time *t* after unloading, *C* is the compliance of the element’s spring, and *b* is the element’s time coefficient (reciprocal of its retardation time, τ). Since during a retarded-elastic recovery the Kelvin elements in a generalized Kelvin model (Figure 1C) all strain independently of one another, post-unloading behavior should be the sum of a set of equations (1) where *i* is a given element’s number and *n* is the total number of elements:

(2) $$\varepsilon (t)=\sum_{i=1}^{n}CL*a_{i}e^{-b_{i}t}$$

and where *C* is now the branch segment’s total retarded-elastic compliance (sum of the compliances of all its Kelvin elements), *a*_i_ is the fraction of that compliance that is associated with element *i*, and *b*_i_ is that element’s time coefficient, the remaining symbols being as in equation (1).

We obtained excellent fits of this form for recovery time courses from nine different species (seven hardwoods and two softwoods), using a curve-fitting computer program that calculated the best-fitting *a*_i_ values for a set of six or seven exponential terms having a range of *b*_i_ values (see Materials and Methods). Supplementary Figure S1 gives an example of recovery time course data fitting by our procedures.

For all of the nine species the component exponential elements’ *t*_1/2_ s were spread over 3–4 decades of the log *t* scale, in all cases extending down to *t*_1/2_s near or below 6 s. These results agree with the previously stated implication, from the data in Figure 6 and Table 1, that branches’ retarded elasticity must involve multiple Kelvin elements with *t*_1/2_s spread over several decades of log time.

### The Retardation Spectrum

For viscoelastic materials generally, and therefore probably for woody branches in particular, it appears that a continuous set of numerous Kelvin elements with successively longer *t*_1/2_s best accounts for their elastic retardation, the several discrete elements with particular *t*_1/2_ values that might best match a retardation time course (as found above) being neither unique nor necessarily qualitatively different, physically, from one another. This continuum of retarded elements is termed a retardation spectrum. In defining it, viscoelastic theory (Findley et al., l976/1989; Ferry, 1980; Christensen, 1982/2003; Kaschta and Schwartzl, 1994, and refs. there cited) replaces equation (2)’s fractional compliance terms *a*_i_ with a retardation-spectral compliance function, here denoted ρ(τ), which is integrated over a range of retardation time corresponding to the extent of the time course data, time usually being expressed as log_10_ time values:

(3) $$\varepsilon (t)=\int_{i=j}^{k}CL*\rho (\tau )*e^{-t/\tau }d\;(\log\;\tau )$$

where symbols are as in equation (2) except that *j* and *k* are, respectively, the lower and upper log time limits of the time course data, and the coefficient *b*_i_ is replaced by 1/τ, the reciprocal of the retardation time (τ) of any component infinitesimal Kelvin element, whose fractional compliance per unit of log retardation time is ρ(τ).

**FIGURE 8:**
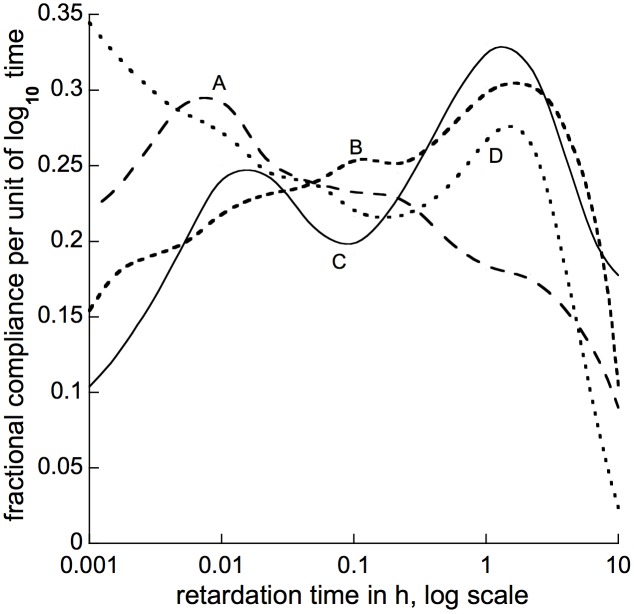
Approximate retardation spectra calculated for bending of branch segments from **(A)** *Liriodendron tulipifera*, **(B)** *Morus alba*, **(C)** *Pseudotsuga menziesii*, and **(D)** *Metasequoia glyptostroboides*. The plotted lines are smooth curves drawn, by computer, through calculated derivative points 2.15-fold apart, spread over the abscissa’s entire time range. These points were obtained from best-fit curve-fitting equations as described in the text, but (for clarity) are omitted from this figure. Spectra obtained for additional species are shown (along with the calculated points on which they are based) in Supplementary Figure S1. Abscissa values are τ in h, plotted on a log_10_ scale.

To accurately deduce a ρ(τ) function from a retarded-elastic recovery time course ε(*t*) is mathematically complicated (Christensen, 1982/2003, and refs. last cited above), and generally involves higher-order derivatives (*e.g.*, Bazant and Xi, 1995) for which “static” time course measurements like the present ones are not sufficiently precise. However, Christensen (1982/2003), p. 69 showed that a simple formula (his equation 4.71) like the following can be used to at least roughly calculate ρ(τ)s from ε(*t*) values:

(4) $$\rho \;(\tau )=d\varepsilon \;(t)\;/\;d\;(\log_{10}\;t)$$

(the *α* term in Christensen’s equation 4.71 disappears when differentiation is by the log of *t*, as here, instead of by *t* itself as in his equation). This type of approximation has been used previously to calculate stress-relaxation spectra (Cosgrove, 1989, and refs. there cited). Following this formula we used computer-calculated log-time derivatives of our above-described curve-fitting equations to obtain approximate retardation spectra for branch bending, 4 of which are shown in Figure 8. Spectra for 5 other species whose recovery time courses we analyzed (Supplementary Figure S2) mostly resembled one or another of those in Figure 8. All but one had a peak between 1 and 10 h of retardation time, and either a second peak around 0.01 h, or a relatively flat shoulder over the intervening range of retardation time.

We are now in a position to examine irreversible branch bending.

### Time Course of Irreversible Bending

The type of irreversible bending of branches that we refer to as permanent set occurs upon an initial, brief application and removal of a load. If the same load is briefly re-applied it causes no additional strain. These features imply that permanent set is time-independent, the usual physical meaning of the term permanent set (*e.g*., Rebouah and Chagnon, 2014).

Turning to the time-dependent irreversible bending that occurs during creep under a steady load, according to rheology’s superposition principle (Findley et al., l976/1989) this creep is the sum of the retarded-elastic and irreversible bendings if they could be followed separately. Therefore, to get the time course of irreversible bending one should subtract, from the post-instantaneous deflection at any given time point during creep, the amount of retarded recovery that occurred at that same (now post-unloading) elapsed time in the branch’s recovery curve like Figure 6, since this represents the time course of retarded-elastic bending unaccompanied by any irreversible bending.

Application of this principle to branches depends on the timing characteristics of their retarded-elastic behavior being unchanged between the post-loading period of creep and the post-unloading period of retarded-elastic recovery. While this seems generally to be true for artificial polymers, it can be verified for the biological material of branches only at the level of mutual consistency between the creep and the strain-recovery curves. Consistency here means at least that the recovery’s time course must involve no more deflection, in any period, than the creep’s, and just as much less deflection as the irreversible bending deflection that remains after recovery is completed (provided that no other bending-affecting conditions, such as possibly a freezing temperature or some biological action on bending, occur prior to the end of the recovery period). We found this consistency to be very generally true of branch bending, but with a rare type of exception noted below.

**FIGURE 9:**
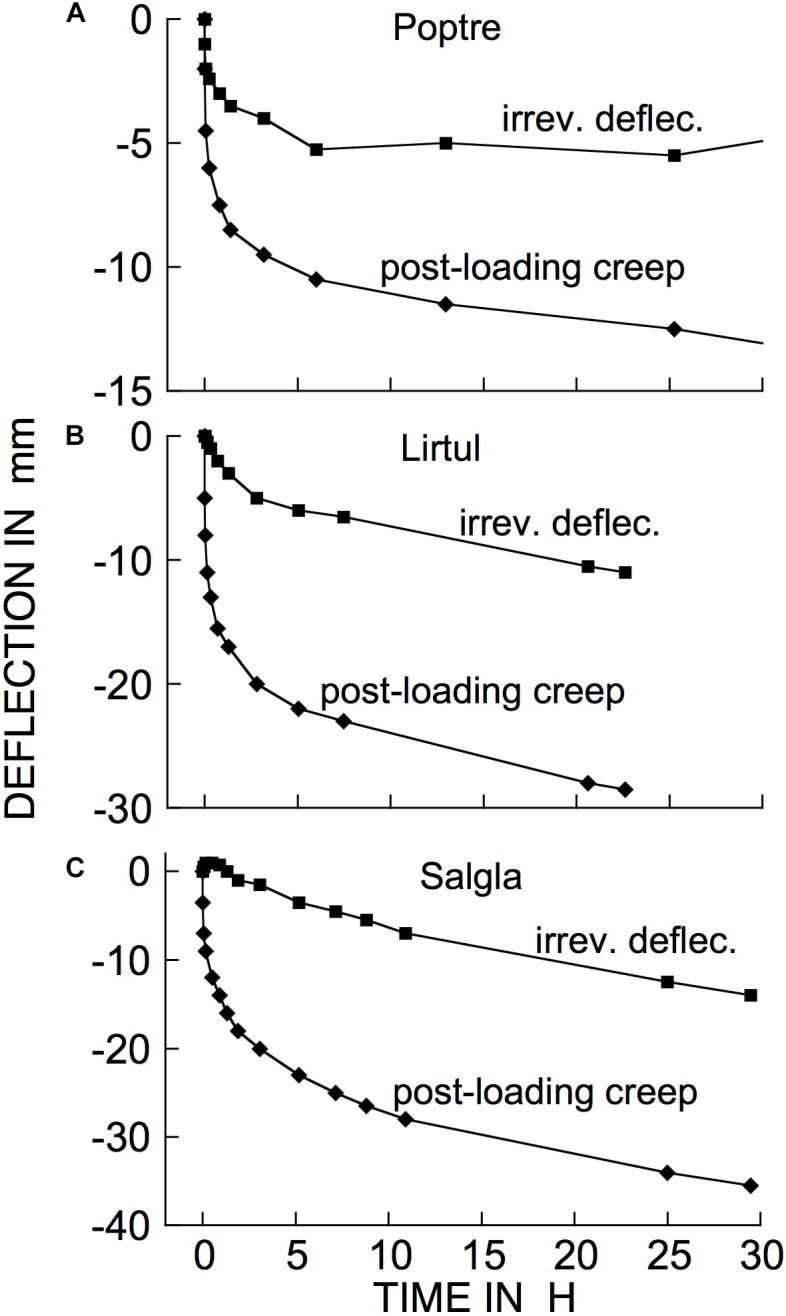
Time courses of irreversible bending (upper curves in each panel) for three species, deduced by subtracting, from the post-loading creep curve (lower curve in each panel), the progress curve for post-unloading retarded recovery (difference between the two curves). Plots begin with the first data point after loading (therefore do not show the instantaneous deflection). **(A)** *Populus tremuloides* (“Poptre”); **(B)** *Liriodendron tulipifera* (“Lirtul”); **(C)** *Salix glauca* (“Salgla”).

**FIGURE 10:**
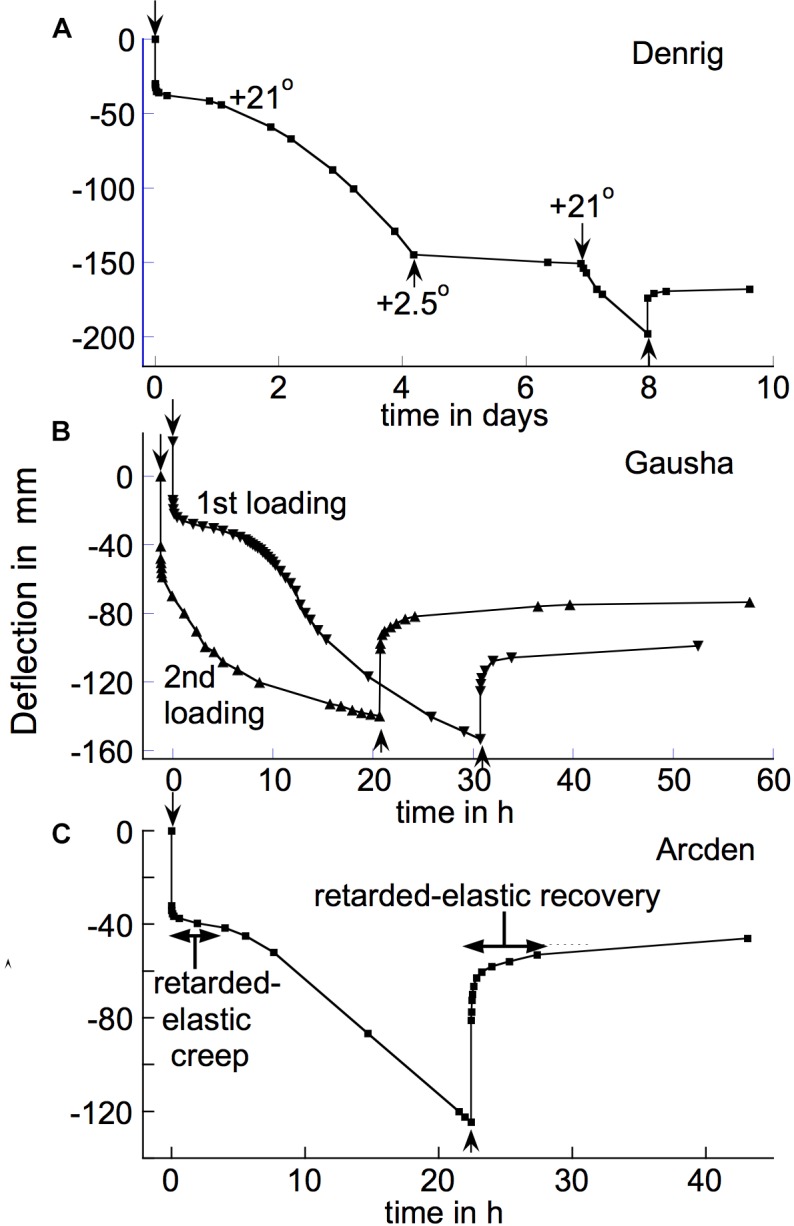
Delayed-onset, long-term irreversible bending of branch segments of **(A)** the shrub *Dendromecon rigida* (“Denrig,” load 234 g); **(B)** the shrub *Gaultheria shallon* (“Gausha,” load 87.7 g); and **(C)** the shrub *Arctostaphylos densiflora* (“Arcden,” load 120 g). Stem segments were loaded or unloaded, respectively, at the ↓ or ↑ arrows. In **(A)**, *T* was changed from +21 to +2.5°C for one period, during which the bending rate was severely reduced. **(B)** After completing its post-unloading recovery from the initial loading, the specimen was re-loaded with the same weight (“2nd loading”) which immediately caused a rapid creep similar to that which developed only after about 8 h beyond the initial loading. **(C)** The increase in retarded-elastic compliance that occurred during the period under load is quite conspicuous, but is also visible in **(B)**. A large fraction of the creep deflections in these graphs was irreversible, as shown by the limited extent of their post-unloading recoveries.

Figure 9 gives examples of the kinds of irreversible-bending time courses that we found for different branches, using this method. In what follows, the “times” numbers within parentheses give the number of times that each of these different patterns was encountered among branches from different species. These patterns included irreversibly bending rapidly just after loading, but declining within a few h either to (Figure 9A) a zero rate (5 times), or to (Figure 9B) a lower, non-zero rate that remained approximately steady out to 24 h (6 times); and (Figure 9C) irreversibly bending almost linearly with time from the start of the loading test, the rate only gradually declining (5 times). With respect to irreversible bending the branches in Figure 9C were apparently behaving, like that in Figure 5C, like a generalized Burgers model (Figure 1B, amplified as in 1C). One further temporal pattern of time-dependent irreversible bending is noted below (Figure 10).

When branches are subject to a loading/unloading cycle that is repeated one or more times on subsequent days, time-dependent irreversible bending similar to, or sometimes even greater than, that which occurred during the first cycle often recurs in subsequent cycles. Therefore, the capacity for time-dependent irreversible bending, which according to Figure 9 usually declines or disappears during a first period of loading, can reappear, over time, during a post-unloading period of a day or more. A slow, possibly biological, process that gradually generates capacity for irreversible bending seems to be operating, in addition to a process that apparently increases steady-flow viscosity relatively rapidly while irreversible bending is occurring. This is important to its biological significance, as noted later.

### Long-Continued and Delayed-Onset Irreversible Bending

With a few species, substantial bending creep continued for long beyond 24 h after loading. This was clearly due to irreversible bending. Figure 10A gives an example, with the poppy family shrub *Dendromecon rigida.* This plot is also noteworthy in that the early part of this time course suggests that substantial irreversible bending began only after about 24 h, when an initially occurring, retarded-elastic deflection had, as usual, apparently become completed. We encountered this delayed-onset irreversible bending in two other species, the Ericaceous shrubs *Arctostaphylos densiflora* and *Gaultheria shallon*. We found it in multiple branches of each of the three species just mentioned.

Figure 10B, illustrating this effect in the last-named species, shows that when the specimen was re-loaded (after it had recovered from its initial loading) it experienced rapid irreversible bending from the outset. Thus the decrease in its resistance against irreversible bending that evidently occurred several h after it was first loaded, evidently persisted through at least a short, subsequent unloaded period.

A similarly long-continued, conspicuous irreversible bending, but without the delayed-onset feature, occurred with branches of the shrub *Forestiera neomexicana* and the ash tree *Fraxinus velutina.* Their post-loading creep resembled that in the “2nd loading” curve in Figure 10B, except that their rapid creep continued for considerably longer (several days) than is shown in that figure. However, with all these species the creep rate eventually declined, so was not a simple steady-flow deformation at constant viscosity.

With at least two of the delayed-onset species their post-unloading retarded recovery involved upward deflections that substantially exceeded the downward deflections that occurred at equivalent times during the initial, post-loading period of creep. This occurred in all 10 branches of *Gaultheria shallon* that we tested. It can be seen by comparing the deflections during the first 2 h of the post-loading and the post-unloading portions of the “1st loading” time course for this species (Figure 10B), and more conspicuously by a similar comparison in the bending/recovery time course shown for *Arctostaphylos densiflora* (Figure 10C). With these species, compliances of Kelvin elements that govern retarded elasticity evidently increased during the initial period under load, possibly at the time that rapid irreversible bending began. This apparently violates the subtraction rule, applied above, for determining the time course of an irreversible bending that occurs during post-loading creep. However, it does not invalidate this rule for the vast majority of tested species, including those in Figure 9.

### Load Dependence of Elastic Bending

**FIGURE 11:**
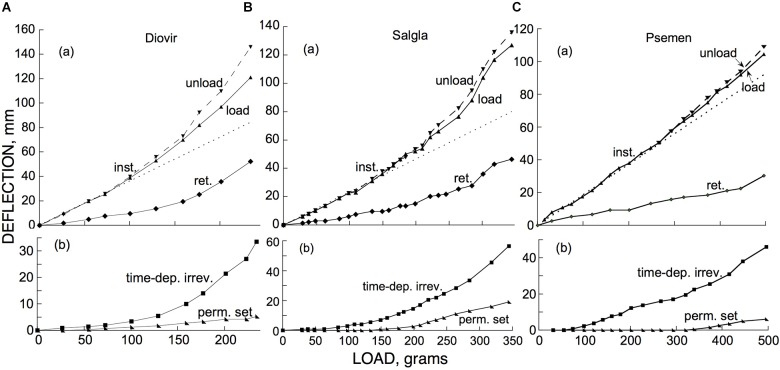
Dependence, on cantilever load, of **(a)** instantaneous (“inst.”) and retarded (“ret.”) elastic bending, and **(b)** permanent set (“perm. set”) and time-dependent irreversible (“time-dep. irrev.”) bending, of branch segments (with lengths × midpoint diameters in mm) of **(A)** *Diospyros virginiana* (“Diovir,” 300 × 6.5), **(B)** *Salix glauca* (“Salgla,” 270 × 6.6), and **(C)** *Pseudotsuga menziesii* (“Psemen,” 350 × 8.0). Both negative (downward) and positive (upward) deflections are here plotted as positive numbers. In panels **(a)** deflections are those that occurred at each of the plotted loads, “load” being the deflection upon loading and “unload” that upon unloading, plotted together so that deviations between them at higher loads can be easily seen, and “ret.” being an estimate of the retarded-elastic recovery that would have occurred if both post-loading retarded-elastic bending and post-unloading (of the given load) recovery had gone to completion, as calculated from the recovery that actually occurred during the 30 min-long post-unloading period as explained in Materials and Methods. In the **(b)** panels, deflections shown are cumulative, *i.e.*, the total of all the deflections of the given kind that had occurred up to and including each given plotted load. Time-dependent irreversible bending’s cumulative deflections extend up to the end of the 30 min period of loading by the weight in question. Dotted lines in **(a)** are straight lines that fit the Hookean (linear) part of the instantaneous response to loading and are extrapolated into the non-Hookean region so that the deviation of the data from linearity can be easily visualized. Data similar to these were obtained, respectively, from a second branch each of *P. menziesii* and of *S. glauca*, as well as from a branch of the shrub *S. pulchra.*

To complete a description of the kinds of bending distinguished above we need to know how each of them depend on the applied bending load. The upper (a) panels of Figure 11 show the effect of imposed loads on the instantaneous and retarded components of elastic bending of branches of two hardwoods (Figure 11A,B) and one softwood (Figure 11C). Both of these components conformed quite well with Hooke’s Law (strain proportional to stress, or load) up to a deflection of about 40 mm (for branch segments about 30 cm long). Over this range of loads and deflections the instantaneous and retarded components of elastic bending occurred in nearly constant proportion to one another as the load was varied.

Above about 40 mm of deflection, concave-upward deviations from Hooke’s Law (increases in compliance) occurred (least conspicuously with the softwood, *Pseudotsuga menziesii*). At these higher loads the ratio of retarded to instantaneous deflection also tended to increase noticeably, at least with the hardwoods [(a) panels in Figure 11A,B].

Whereas in the linear (Hookean) range the instantaneous deflections upon loading and upon unloading were equal (as expected; unloading deflections were the negative of the “unload” numbers plotted in Figure 11), with both of the hardwoods in the non-Hookean range of deflections, the instantaneous recovery upon unloading exceeded the branch’s previous instantaneous deflection upon that load’s application. This difference increased as the total load was further increased [(a) panels in Figure 11A,B]. This apparently represented an increase that occurred during each of these 30 min periods of loading, in the specimen’s instantaneous elastic compliance. This was confirmed by subsequently reloading the specimen with a smaller load, within the Hookean (linear) range. This caused an immediate deflection that exceeded what had occurred earlier at this load, by about the same percent as the post-loading increase in deflection that had occurred at the highest load (in the non-Hookean range) that had been applied up to that time. While the increase in instantaneous compliance resulting from loads in the non-Hookean range correlated well with the non-linear elastic response, the measured increase in instantaneous compliance could by itself explain only part of the elastic response curves’ deviation from linearity in this range of loads.

For the softwood branch that we tested (Figure 11C), an increase in instantaneous compliance during the loading periods similarly appeared at loads in the non-Hookean range but was slight, just as were the deviations of the deflections from linearity with load in that range. A similar progressive-loading experiment on a different branch of this species gave similar results.

### Load Dependence of Irreversible Bending

Figure 11’s lower (b) panels show the extent of permanent set and of time-dependent irreversible bending in the loading experiments just considered. Because of the mostly small individual irreversible deflections that occurred during the short periods of successive loadings, the data are plotted as the cumulative deflection (sum of the successive load-after-load increments) up to and including each of the plotted loads.

In Figure 11, permanent set was negligible (∼1 mm at one or two loadings at most) at loads below that at which non-Hookean elastic behavior began to occur. Above that point it increased more or less linearly with further increase in load. Permanent set, therefore, appears to involve a yield stress (*Y*, a non-zero value of stress below which little or no irreversible strain occurs, *cf*. Barnes, 1999). When additional load is imposed, the specimen supports it by an irreversible strain that causes an increase in *Y*. For all 3 branches in Figure 11 the increase in *Y* above its initial value was at least approximately proportional to the bending deflection.

Occasional branches, other than those used for Figure 11, gave several mm of permanent set at the more modest load that was to be used for one of the types of experiment considered above (involving an instantaneous deflection of 30–40 mm). It is unclear whether these were instances of a much lower initial *Y*, or involve a mechanism different from that of the permanent set plots in Figure 11, but as noted by Barnes (1999), for many solids *Y* is often less sharp or definite than appears in the present plots.

In contrast to permanent set, in these tests time-dependent irreversible bending occurred progressively from, or (in Figure 11B,C) from nearly, the smallest applied loads. It therefore appears, in comparison to permanent set, to involve relatively little, or no, yield stress. Since the slope of the plot, at any point in a cumulative plot of rate vs. load, rather than the rate itself at that point, relates to the process’s dependence on load, the concave-upward form of these plots indicates that the irreversible bending rate increased progressively with load at least roughly linearly.

## Discussion

Our results show that, under cantilever loads, both retarded and instantaneous elastic bending, as well as two kinds of irreversible bending, are of general occurrence in woody branches. Although we found large quantitative variations, in viscoelastic bending, throughout the range of species tested, one apparently consistent difference that our data indicate is a significantly larger average viscoelastic relative to instantaneous elastic compliance in hardwoods (Angiosperms) compared with softwoods (Gymnosperms). This might seem surprising, considering that the xylem of hardwoods, as their name implies, is stiffer and tougher than that of softwoods. The greater retarded-elastic compliance of hardwoods might be due to the presence, in the wood, of cells such as vessel members, fiber-tracheids, and/or abundant xylem parenchyma that are missing in typical softwoods.

As mentioned in the Introduction, we have encountered almost no descriptions of the retarded elasticity of branches in the botanical literature. However, Alméras et al. (2002) demonstrated bending creep of apricot tree branches using the simple method illustrated by our Figure 4. Their Figure 7 did not distinguish instantaneous elasticity from the important first 10 min of creep, nor did they report testing, by subsequent unloading, whether the branches had experienced irreversible as well as retarded-elastic bending (this seems likely).

Because the elasticity of even a small, woody, non-tropical branch (∼6 mm in diameter) is due, we found, mainly to properties of its xylem, it not surprisingly relates to wood’s known elastic properties. Retarded elasticity, stress relaxation and irreversible straining of wood have long been known (Kitazawa, 1947; Grossman, 1954; Grossman and Kingston, 1954; Youngs, 1957; Kollmann and Coté, 1968). However, viscoelastic behavior is a relatively minor component of the compliance of commercially used, air-dry wood (usually ∼12% moisture), as compared with wet wood (Youngs, 1957; Kollmann and Coté, 1968; Schniewind, 1979; Dinwoodie, 2000; Hogan and Niklas, 2004) or with living, well-hydrated branch segments such as those in the present work, around 50% of whose fresh weight is water.

### Features of Woody Branches’ Bending Elasticity

For the branches we tested, both instantaneous and retarded-elastic deflections were proportional to cantilever load (*i.e.*, followed Hooke’s Law) up to a deflection of about 40 mm (for branch segments of the dimensions we used; Figure 11). Above this, elastic deflection increased more than in proportion to load, *i.e.*, the elastic compliance progressively increased (the xylem secondary walls’ tensile moduli decreased), with the retarded compliance increasing even more than the instantaneous.

For the tested hardwoods, the slope of the elastic strain/load curves in the non-Hookean range of loads increased by more than 50% above that in the Hookean range (Figure 11). This is much greater than the increase in compliance that we obtained from stripping the bark from woody branch segments of the size that we used. Therefore, it appears that the non-Hookean behavior at higher loads is mainly a property of the secondary walls of xylem cells rather than of mechanical cells in the bark (fibers, cork).

The elastic bending of branches, supported by the thick secondary walls of their xylem, contrasts with the elastic extension of growing primary cell walls, whose tensile modulus increases, often markedly (compliance decreases), with increase in tensile stress imposed either by turgor pressure (Kamiya et al., 1963; Ray and Ruesink, 1963, Figure 3; Steudle et al., 1977; Büchner et al., 1981) or by an externally applied uniaxial load (Cleland, 1967; Thompson, 2001, Figures 1, 3). Thus primary walls become markedly stiffer as elastic strain increases, rather than Hookean at low strains and becoming weaker at higher strains, as in our tests on branch bending. This contrast probably reflects, at least partly, a biophysical difference between primary and secondary walls that results from the relation between wall deposition and cell growth in size, as follows.

Xylem cells deposit their secondary wall while at an unchanging, final (post-elongation) cell size, so all its layers have the same unstretched length. All the layers therefore begin to bear stress as soon as any strain occurs, so the modulus, at least over a certain range of strain, will remain constant (Hookean straining). Their concave-up, non-Hookean straining at higher stresses, as in branch bending studied here, presumably results from a weakening or loss of some interpolymer-chain bonds under these stresses. This loss is indicated by the increase in instantaneous compliance that occurred during exposure to non-Hookean loads (Figure 11).

In contrast, while a cell is growing, each cell wall layer successively deposited at the plasma membrane surface (Ray, 1967) must have, at the time it was deposited, an unstretched length equal to the cell’s length at that time, therefore longer than that of previously deposited layers. As the cell elongates, the then-existing layers become stretched irreversibly, for which they must acquire and retain some stress by post-depositional wall extension, stress which means that their unstretched lengths remain shorter than that of the wall’s most recently deposited (stress-less) layer. Because of the loose structure and thinness of the primary wall, it appears that its layers scarcely support any compressive stress (indicating this are wrinkles or folds often visible, in electron micrographs of relaxed primary walls, on their inner [greatest unstretched length] surface, *e.g.*, Roland et al., 1982; Evered et al., 2007, Figure 4a). This tends to make the primary wall’s elasticity non-Hookean, because as it is extended, starting from a relaxed state, its older, shorter, unstretched layers are at first the only ones that experience significant tensile stress, whereas its more recent layers begin to bear significant stress only as the cell’s overall length is increased beyond these layers’ unstretched lengths. This progressive increase in the fraction of the cell wall’s cross section that bears substantial tensile stress results in the wall’s modulus progressively rising with strain.

Another factor probably contributing to the elastic difference between primary and secondary walls is that secondary walls’ microfibrils run essentially straight (*e.g.*, Dinwoodie, 2000; Figure 1.18), and therefore should undergo strain and experience stress at even the smallest cell wall extensions. In contrast, electron micrographs of primary walls (Gunning and Steer, 1996; Zhang et al., 2013) show that their microfibrils tend, when the wall is in a relaxed state, to run somewhat sinuously in the plane of the wall, so will start to undergo tensile strain and thus bear significant stress only after they have become straightened by a certain amount of preliminary wall strain.

Spatz et al. (1999) proposed that because a secondary wall’s non-cellulosic matrix’s elastic modulus is much lower than that of its microfibrils, upon unloading a wall that had been stretched to its yield point the matrix will tend to go into compression while the microfibrils remain under tension. This allows, they argue, the wall to have Hookean elastic behavior, which our work confirms for woody branch bending. A growing primary wall’s matrix, however, has a much higher water content and thus a much lower polymer density, so can be expected, as stated above for the primary wall as a whole, not to significantly resist, or support, compression. When compressed its matrix polymers probably collapse into the normally water-filled, capillary interstices between them, and its microfibrils probably contract below their unstretched length with little or no compressive stress by going into a sinuous course through the matrix. This will allow an unloaded primary wall to shrink essentially to the unstretched length of its shortest unstretched layer(s), resulting in strongly non-Hookean elasticity, for the reasons explained above, when a tensile load is imposed on it.

### Characteristics of Branches’ Retarded Elasticity

Our retarded recovery time course data imply that multiple Kelvin elements contribute to the retarded portion of branches’ elasticity, so a generalized Kelvin model (Figure 1C, with many Kelvin elements), or (better) a retardation spectrum, is needed to describe fully this behavior. In contrast, Thompson (2001, 2007) analyzed extension creep of tomato fruit and sunflower hypocotyl primary-wall skeletons as comprising only 2 Kelvin elements, but including also a log-time component that could have been composed of multiple Kelvin elements.

We calculated approximate branch-bending retardation spectra (Figure 8 and Supplementary Figure S1) from our retarded-recovery curve-fitting equations, entered into equation (4). The spectra extended over about 4 decades of log time, with a tendency for (usually) modest peaks in the vicinity of 0.01 h (36 s) and 1 h. The differences in τ values across the spectrum could be due to differences in either spring compliance (*C*) or in dashpot viscosity (η), or both, between different adjustment mechanisms, since τ = *C* ^∗^η (or as more often written, τ = *η/E*, where *E* is an element’s tensile modulus). Two-peaked relaxation spectra of artificial polymers, somewhat like some of our retardation spectra, have been interpreted as due primarily to differences in effective viscosity involved, respectively, in local movement of short polymer chain segments (short relaxation times) as against the long-distance displacement of complete chains or of long chain segments between cross-links or “entanglement coupling” points (long relaxation times) (Ferry, 1980). Whether something similar applies to plant secondary walls might be found by an approach similar to that of Macmillan et al. (2013).

Our retardation spectra indicate substantial compliance extending down essentially to the zero-time lower limit of about 3 s for our “static” time course measurements. This implies that branches’ complete retardation spectra probably extend down into a time range well below 3 s, a range that is inaccessible by our measurements but has been found for a wide range of viscoelastic materials using “dynamic” measurements of stress as a function of oscillating strain (Findley et al., l976/1989; Ferry, 1980; Hansen et al., 2011). Dynamic measurements on wood (Dunlop, 1978; Ouis, 2002, and refs. there cited) revealed damping elements with *t*_1/2_s extending down into at least the millisecond range. And tweaking the tip of a small branch segment clamped as in our measurements starts an elastic oscillation that damps down to zero within about 1–2 s (depending upon species), suggesting substantial retardation in the sub-second *t*_1/2_ range. Retardations in this time range should dissipate extra energy to the same degree as slower, conventional retardations do, so they become significant in interpreting retarded elasticity’s biological significance as noted below.

### Characteristics of Irreversible Bending of Branches

Branch irreversible bending consisted of two components somewhat analogous to instantaneous and retarded elasticity, namely, “permanent set” that occurs immediately when a load is imposed, and time-dependent irreversible bending, which occurs subsequently. In the branches that we tested for dependence of bending on load, permanent set became substantial only when loads in the elastically non-Hookean range were imposed. A substantial yield stress (*Y*) is thus associated with it, so it is at least somewhat analogous to a *plastic* deformation (in the sense of plasticity’s meaning in the rheological literature: Findley et al., l976/1989; Jaeger, 2012). This type of bending may be analogous to irreversible straining of wood above a *Y* as reported by Köhler and Spatz (2002) and Keckés et al. (2003), in which (unlike in classical plasticity) stress increased progressively with strain above the yield point (“strain hardening”). This behavior was apparently instantaneous (like our permanent set) or nearly so, since it was detected by brief Instron stretching experiments, which primarily measure instantaneous or very short-term deformations. It may also be related to the “first plastic” phase of wood cell wall yielding as molecularly modeled by Jin et al. (2015), since this phase involved a yield stress and strain-hardening.

Pao and Marin (1953) and Youngs (1957) (pp. 88–89 and his Figure 42) clearly enunciated, and Engelund and Salmén (2012, their Figure 3C) later used, the creep-minus-recovery time course subtraction principle that we independently inferred, for determining the course of time-dependent irreversible straining during a creep experiment. Youngs’ (1957, Figure 43) data on irreversibleon wood formation by the c straining of red oak wood revealed a rate that progressively declined with time, as in most of the branches tested here (*e.g.*, Figure 8), and involved a definite *Y* (at least at 80°F). Two of the branches we tested [Figure 10B,C (b) panels] showed a narrow range of load, up to about 50 gm, over which little or no time-dependent irreversible bending occurred, and above which the strain rate increased in a concave-up manner as would be expected (since cumulative irreversible strain was plotted) if strain rate depends at least approximately linearly on load [as did that of red oak wood, Youngs (1957)]. Time-dependent irreversible branch bending thus seems at least roughly analogous to a viscoplastic or “Bingham” deformation, as with the irreversible extension of primary walls during cell growth (the Lockhart equation: Cosgrove, 2016, Figure 1), but with the viscosity (η) controlling it usually increasing with time, and the yield stress apparently small (or sometimes practically 0) compared to that for either permanent set in branches, or for primary wall irreversible extension.

Cosgrove and Jarvis (2012) give a broader review of secondary wall biomechanics, beyond the points needed for interpreting the results of our branch bending measurements.

**FIGURE 12:**
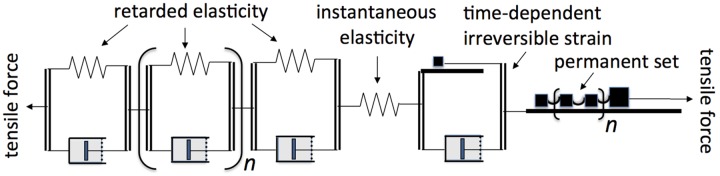
Rheological model representing the viscoelastic bending behavior of shrub and tree branches, according to present results. Thick horizontal lines (part of each St-Venant element, named after the French physicist Adhémar de Saint-Venant who in 1870 published the first general equations for plasticity, *cf*. Chen and Han, 2007) represent rough surfaces upon which blocks (black squares) rest, exerting static and dynamic friction (the yield stress, *Y*). The multiple blocks in the “permanent set” St-Venant element are coupled loosely together so that all but the first block come into action only after strain has straightened the coupling between any one of them and those ahead of it, causing *Y* to increase progressively with strain. The *n* for retarded elasticity and permanent set elements implies a large number, respectively, of Kelvin elements creating a continuous retardation spectrum, and of friction blocks creating an essentially continuous rise of *Y* with strain.

For a rheological model that agrees with the behavior of branches that exhibit irreversible bending we have to add, to the generalized Kelvin model for retarded elasticity (Figure 1C), two elements in series with it, one for permanent set and the other for time-dependent irreversible bending (Figure 12). Each of these involves a yield stress (*Y*) represented by a “St-Venant” element (Jaeger, 2012, p. 100) which depicts *Y* as the static and dynamic friction (assumed to be equal) between a block and the surface upon which it is resting. The single, small block in Figure 12 represents the relatively small *Y* that may be involved in (at least some) time-dependent irreversible bending, the dynamics of which involve mainly the dashpot’s viscosity (η). The multiple blocks coupled loosely together in the “permanent set” element [a “generalized St-Venant element,” *cf*. Jaeger (2012), Figure 35(d)] provide that as strain increases and the couplings progressively become stretched tight, the total frictional resistance, and thus *Y*, increases (“strain-hardening”), as seen in our Figure 11 “perm. set” plots. The *n* value for Kelvin elements in Figure 12 has to be at least 6, from our analysis of retarded-elastic recovery, and is probably much larger (for a continuous retardation spectrum). The same probably holds for the multiple St-Venant friction-block elements representing permanent set, since its strain-hardening appears to be a continuous increase in *Y* with increase in load (Figure 11).

The generally encountered decline in branch irreversible bending rate over time implies a time-dependent decrease in its steady-state η or increase in its *Y*, or both of these. One possible way this could occur would be by an onset of reaction wood formation by the cambium (*cf.* review by Felten and Sundberg (2013)), since reaction wood acts to oppose stem bending.

### Long-Continued and Delayed-Onset Irreversible Bending

We encountered a few species whose branches underwent substantial irreversible bending for much longer than 24 h after being loaded. These species included three whose irreversible bending began only belatedly subsequent to loading (Figure 10). Either the η governing its rate, or an unusually high *Y* preventing its initial occurrence, evidently decreased markedly at a time when the post-loading, retarded-elastic bending was nearly complete. This is not normal for viscoelastic material, but might be at least remotely related to the “thixotropy” of certain gels such as yogurt, which become less viscous above a certain level of stress (Barnes, 1997). Or the decrease in η or *Y* might instead be caused biologically, since these were living specimens. It is especially curious that in at least 2 of these species, retarded-elastic compliance clearly increased remarkably along with the time-dependent decrease in resistance to irreversible bending (Figure 10B,C).

### Biological Roles of Retarded Elasticity

The retarded elasticity of tundra shrub branches largely explains why their springtime recovery of elevation after becoming unloaded of snow (Sturm et al., 2005) is gradual, rather than entirely immediate. Retarded elasticity also, by increasing the downward inclination of snow-loaded branches beyond that obtainable instantaneously, should increase the ability of trees and shrubs to shed snow, as well as decrease their interception of snow (Pomeroy and Goodison, 1997), above what can be inferred from instantaneous or short-term bending moduli (Schmidt and Pomeroy, 1990, and refs. there cited).

More generally, retarded elasticity of shrub and tree branches might reduce the hazard of wind gusts that tend to break them. This might be analogous to the function of vehicle shock absorbers^2^, which utilize retardation to slow the displacement of the vehicle’s suspension elements during and after it encounters a bump, reducing their maximum displacement and reducing or eliminating the vehicle’s subsequent, bothersome elastic rebound.

By being in series with an unretarded (“instantaneous”) elasticity, the principal retardation that our branch measurements reveal cannot dampen much of branches’ elastic bending the way an automobile shock absorber works, where spring elasticity is entirely in parallel with retardation (as in a single Kelvin element). However, two ways can be identified by which retarded elasticity may protect branches from wind-gust breakage.

During a windstorm, gusts typically last about 5 s (Sellier and Fourcaud, 2009). Since most of branches’ retarded elastic compliance seen in our measurements involves *t*_1/2_ values much longer than 5 s (Table 1), that fraction of their retarded compliance undergoes little strain over periods of up to 5 s. Retardation thus limits elastic bending during gusts to less than about two-thirds of what the branch’s total compliance would allow if none of it were retarded. It thus reduces, perhaps importantly, the chances that the branch gets bent beyond its breaking strain during a gust.

A second way in which retardation probably helps protect branches from wind-gust breakage depends upon our conclusion (reached above) that branches’ retardation spectra likely extend down significantly to short-*t*_1/2_ elements within what our static method of measurement treats as “instantaneous” elasticity. Since such elements will get flexed to, or nearly to, their elastic equilibrium point during a bending of as brief as 5 s, the energy that their viscous action dissipates (which can exceed the energy that they absorb elastically, *cf*. Findley et al., l976/1989, pp. 95–96) should reduce, in a shock-absorber-like action, the kinetic energy that a branch acquires from a wind gust and thus how fast and how far it bends during and after the gust, thereby helping to reduce the chances of breakage.

### Biological Roles of Irreversible Bending

The irreversible bending that we observed (Figure 8) was generally much too slow to appreciably influence the effect on stems of pulsatile forces such as wind gusts. However, this slow, irreversible bending probably allows the time-dependent decline in attitude of lateral branches, and increase in their downward curvature, that one can observe in the crowns of many trees that have an excurrent (“Christmas tree”) over-all crown form, and in some deliquescent (elm-like) crowns with “weeping” branches (*e.g*., weeping willow). These changes are a significant part of the development of tree crown morphology, which is important to aspects of forest canopy behavior such as interception of sunlight and of precipitation (especially snow). The repeated occurrence, noted in Results, of time-dependent irreversible bending (and perhaps also of permanent set) upon later re-loadings of a branch are probably important in allowing these gradual changes in the form of tree crowns to occur.

Irreversible bending is likely to be the basis for the gradual decline, toward ground level, of the older portions of tundra shrub branches, and possibly of some other shrubs, *e.g.*, raspberries (*Rubus* spp.). In tundra, this decline hastens these portions’ engulfment by upgrowth of the tundra’s ground-surface moss layer, after which the engulfed portion of the stem adventitiously roots and becomes part of the shrub’s below-ground absorption and mechanical support system. This process keeps these shrubs’ elevation above the landscape surface more or less constant over time despite the yearly upward elongation of their branch tips, improving the shrubs’ chances for protection, by snow covering, against winter hazards such as low-temperature extremes.

## Conclusion

Retarded elasticity, occurring over a time scale extending usually out to about 24 h beyond the time of loading or unloading, is an important component of woody stem bending elasticity, commonly involving a compliance of as much as 30–50% of the instantaneous bending compliance, especially in hardwoods, and apparently less important (but still present) in softwoods. It involves a wide quantitative range of retarded-elastic (“Kelvin”) elements, with retardation times ranging at least from seconds up to as long as 19 h (depending on the specimen), the form of which distribution (the retardation spectrum) we calculate and display (Figure 8). It probably extends downward into the subsecond or even millisecond range which, by the static measuring method used here, falls within what we record as “instantaneous” elastic strain. Because of their Kelvin elements, branches undergo *stress relaxation* when bent to a certain angle and held thereafter at that angle.

We distinguish two kinds of irreversible bending of branches under cantilever loads: (a) “permanent set” which occurs immediately when an applied load exceeds a yield threshold, and increases with load above that point, and (b) slow, time-dependent irreversible bending, which occurs significantly in many but not all branch specimens, and more rapidly and extensively in a few. Permanent set, which is effectively instantaneous and involves a substantial yield stress that increases with strain, is apparently comparable to a plastic deformation previously reported from Instron extension tests on wood. Time-dependent irreversible bending on the other hand is nominally viscous in nature, its rate increasing with load above an at most small yield stress, but its viscosity usually increasing (in stiffness) with time. However, in a few species, resistance to irreversible bending decreases, some hours after loading, to much below its initial value, causing such bending to begin belatedly. This departs remarkably from typical viscoelastic behavior and theory, and deserves further investigation.

Several features of both elastic and irreversible bending of branches (which are features of their xylem secondary cell walls) contrast with corresponding features of the extension behavior of the primary walls of growing cells. The elasticity differences (secondary walls: Hookean, but convex to the load axis at high loads; primary walls: non-Hookean throughout, strongly concave to the load axis at all loads up to full turgor) are probably due, at least partly, to primary wall deposition occurring over the course of growth in cell size so its layers tend to differ from one another in unstretched dimensions, whilst a secondary wall is deposited at an unchanging cell size so all its layers have the same unstretched dimensions. Differences between primary and secondary walls in the straightness of their cellulose microfibrils (when unstrained) also probably contribute to their different elastic behavior. The differences in time-dependent irreversible straining (*e.g.*, secondary walls not exhibiting the high yield stress found with growing primary walls) may be due to this being essentially physical in secondary walls but involving a biochemical component [*e.g.*, expansions (Cosgrove, 1997, 2016), or hydroxyl radicals (Schopfer and Liszkay, 2006; Heyno et al., 2011)] in primary walls.

Retarded elasticity has a probable benefit of damping the flailing motions of small branches caused by gusts in windstorms, that tend to break them, causing loss of photosynthetic leaf area and reproductive potential. Irreversible bending probably contributes importantly to the development of the form of tree and shrub crowns, which affects their ability both to absorb solar radiation, and to shed snow without breaking. It is doubtless also involved in the typical growth form of many arctic tundra shrubs, and certain others, that maintain, by downward bending of the older parts of their stems, a more or less constant height above the landscape surface, despite upward annual stem elongation at their branch tips.

## Author Contributions

Both authors contributed to the conception of the project and collaborated in collecting plant material for the assays, interpreting the results, writing the manuscript, and preparing the figures. PR developed the method and made most of the measurements. MB-H also performed statistical analyses.

## Conflict of Interest Statement

The authors declare that the research was conducted in the absence of any commercial or financial relationships that could be construed as a potential conflict of interest.

## Footnotes

1 Synergy Software (2014) KaleidaGraph, Version 4.5.2 (Mac). www.synergy.com

2 Wikipedia (2017) Shock absorbers. https://en.wikipedia.org/wiki/Shock_absorber
