# Supplementary material for: Elastic and Irreversible Bending of Tree and Shrub Branches Under Cantilever Loads
Source: Front Plant Sci. 2019 Feb 5;10:59. doi: 10.3389/fpls.2019.00059 (PMC6370663; doi:10.3389/fpls.2019.00059)
Supplement: Supplementary file 1 [file Presentation_1.pdf]

**Elastic and irreversible bending of tree and shrub branches under cantilever loads**

**Peter M. Ray[1, 2*] and M. Syndonia Bret-Harte[2]**

[1]Department of Biological Sciences, Stanford University, Stanford, CA, USA,

[2]Institute of Arctic Biology, University of Alaska Fairbanks, Fairbanks, AK, USA

ONLINE SUPPLEMENTARY MATERIAL

**Bending assay procedure**

This description is additional to that given in the main paper's Methods section and applies to the latter's Figures 2 and 3. Single capital letters here refer to the labels in Figure 2.

The bar-to-bar clamp (Eisco CH0664JM clamp holder, or the equivalent) that was used to hold the branch segment's basal end had a horizontal, upwardly directed, flat bearing surface 4.5 cm long, backed by a flat, vertical surface with a sharp right angle between them. Its flat-tipped clamping screw descended at a 45° angle toward that right angle, pinning the basal part of the branch segment against that angle. The area of contact between the screw's tip and the branch was covered by a 1.7 mm thick, semi-cylindrical, hard polyethylene sleeve, cut from polyethylene tubing of an inside diameter similar to the branch segment's basal end's diameter. This sleeve (shown in Figure 2 but not labeled) spread the force exerted by the clamping screw over a larger area of branch bark than that of the screw's tip, and prevented penetration of branch tissue by that tip. The clamping method prevented the branch base from twisting or curving within the clamp under the loads that we applied to the segment's tip. Rod C, to which the bar-to-bar clamp was clamped by its horizonal second screw, passed through holes drilled in the bottom and top members of frame D. These holes held rod C rigidly, which, along with the

rod's rigidity and the the branch's clamping method, prevented any deflection from occurring at the branch segment's base when a load was applied to, or removed from, its tip.

Before applying any load, the branch segment tip's initial elevation was marked on the record paper (G), which had been mounted, using small pieces of "scotch" tape at its corners, on a laterally movable plexiglas panel (F). To apply load, a weight (usually a previously weighed bolt) was hung (by a small loop of string) from the branch segment's tip. The load to be used in the experiment was chosen by trials with lighter-than-necessary weights, successively increasing until one that gave (usually) between 3 and 4 cm of deflection was found. This load was immediately removed, and any residual deflection that it (and previous, only briefly imposed lighter weights) had caused (permanent set) was recorded as a second mark on the record paper. The chosen weight was then re-applied, and the immediate (zero-time) downward deflection ("instantaneous" elastic bending) was recorded (along with the time of day) within about 3 s of loading, and similarly, later, for the upward deflection upon unloading (instantaneous elastic recovery). Further deflections (bending creep after loading, elastic recovery after unloading) were recorded at appropriate time points whose values were noted next to each mark.

Some branches' deflection upon or after being loaded or unloaded involved a small horizontal component which was compensated, when it occurred, by slightly swiveling the segment's clamp on its mounting rod to keep the pointer pin's tip near, but not quite touching, the record paper and thereby avoid direct contact, that would inhibit further bending, or excessive parallax in marking the pointer tip's position on the record paper. If the loading weight made contact with the record paper, any friction between the two that might impede deflection was overcome, between position markings, by gently tapping on the bending apparatus' frame (D).

Loaded branches tended to curve fairly uniformly along their length, their apical to basal taper evidently largely compensating for the increase in bending moment toward the base. In the case of occasional branch segments that did not taper noticeably, they curved, when loaded, more strongly near the base than elsewhere, as expected. No branches broke under the cantilever loads that we applied to them.

Over periods of many hours involved in experiments some branch bases would tend to loosen in the clamp by evaporative shrinkage and/or time-dependent compression by the clamping screw. Any such loosening was forestalled by periodically advancing the clamping screw judiciously to maintain the initial clamping strength.

After a time interval of normally about 24 h under load, by which time bending creep usually had (or had virtually) ceased, the movable plexiglas panel (F) bearing the record paper (G) was moved horizontally (arrows in Figure 2) behind the branch segment by about 20 mm and a new position mark was recorded, so that the record of upward deflections resulting from unloading and/or other experimental treatments would not overlap with the downward deflection record. Before and after unloading, pointer-pin tip position marks were then recorded until upward deflection (recovery) ceased to occur, almost always within about 24 more h. At that time the branch segment's basal clamping screw was tightened snugly once more, in case any belated shrinkage that might slightly loosen the segment in its clamp had occurred since the last previous tightening. After recording the pointer tip's final position, any deflection (below the initial, post permanent-set elevation) that then remained was considered time-dependent irreversible bending.

In later experiments, especially the species-survey ones, two branch segments were mounted together on the apparatus, using separate clamps both attached to its rod (C), so that double the

amount of data could be collected in each run. The lower of the two segments was cut about 5 cm shorter than the upper one, so that the weight hanging from the upper one's tip would not block recording the positions of the lower one's pointer tip, and their respective records would not overlap. For the lower, shorter and usually thinner branch segment, which usually needed only a relatively light load, we sometimes used a smaller clamp (Precision Scientific 59-5290 clamp holder) which had, like the abovementioned Eisco clamp, a 45$^{o}$ clamping screw and horizontal bearing surface but the latter only 2.7 mm long, and whose dimensions allowed it to be raised relatively closer to the upper clamp (than an Eisco clamp could) so that the lower branch segment could undergo a normal amount of downward deflection without its weight contacting the basal frame of the apparatus, precluding it from completing its creep.

For comparing retarded-elastic or irreversible bending between different species or treatments, deflections in mm were normalized by dividing by the initial, "instantaneous" elastic deflection from the same run. For calculating mean values for different species, to be used to obtain mean values for different biomes or for comparing different growth forms such as hardwoods *vs*. softwoods, the mean of two different calculations from each bending test was used. Retarded elastic bending was calculated either (1) from the deflection that occurred during the post-unloading recovery period, or (2) as the difference between the post-loading creep deflection and the irreversible bending deflection that occurred during the period under load, calculated as stated in (3) next. Irreversible deflection was calculated either (3) as the difference between the total (downward) deflection during the period under load and the total (upward) deflection that occurred during the post-loading recovery period, or (4) as the difference beween the creep that occurred while the specimen was under load and the retarded recovery that occurred during post-unloading period. All these deflections were normalized to the initial,

"instantaneous" elastic deflection as noted above. The rate of irreversible bending was obtained by dividing the thus-normalized irreversible deflections by the number of hours that the specimen had been under load.

The method for calculating the retarded and irreversible deflections in the experiments on load dependence (Figure 11) is given in the main paper's Methods.

The fraction of the cantilever load that acts to cause bending, as against elongation, of a stem segment decreases below 1 progressively as the angle of the branch segment's tip below the horizontal increases. Therefore, the deviation of the bending curves from linear (Hookean) form, in Figure 11, at the higher loads (greater deflections) is actually greater than appears in the plots. We have not attempted to correct for this effect in any of our plots (they assume a constant bending load), because the tip's angle from the horizontal varied, relative to its deflection, depending upon how curvature, during bending, was distribued along the segment's length, which we do not know for any particular stem segment and can differ between different segments depending upon their respective distributions of taper and of elastic modulus.

**Computer curve-fitting of retarded recovery time course data**

The curve-fitting computer program of Kaleidagraph[1] was used to calculate, for each recovery time course, the best-fitting $a_i$ values for a set either of 4 (Method 2), or of 6 or 7 (Method 1), exponential terms for an equation of the form

$$\varepsilon(t) = \sum_{i=1}^{n} CL * a_i e^{-b_i t}$$

whose $b_i$ coefficients either were initially specified (Method 1) or were chosen by the computer (Method 2) as described below. Best-fitting means the closest possible approach, over-all, of the calculated curve to the recovery data points. To place the zero-time (moment of unloading) point of retarded recovery data into the log time plot that was used for these calculations it was

assigned a value of about 3 s, and this amount was added to each of the subsequent time points' nominal values, which significantly affected only (and only slightly) the position of the second point (usually at about 30 s) in the plot.

Figure S1 shows examples of our two methods of curve-fitting, for one particular recovery time course. In Method 1, $b_i$ coefficients for 6 exponential terms were initially chosen, spread evenly (on a log scale) over the time range of the recovery data, and the computer calculated the $a_i$ coefficients for these terms that would give the best possible fit[1]. If, as in Figure S1A, the initially calculated curve failed to pass through some of the data points, by trial and error the operator would adjust some of the $b_i$ values slightly (and/or add a 7$^{th}$ $b_i$ coefficient) and have the computer repeat the calculation, continuing iteratively until the calculated curve passed through, or at least touched, as many of the data points as possible (Figure S1B).

In Method 2, the computer calculated both the $a_i$ and the $b_i$ coefficients for each term but the equation was restricted to only 4 exponential terms, because 4 bivariate terms were the maximum that the curve-fitting program could handle. Repeating the calculation, as in Method 1, in this case would not improve the perfectness of fit. Fits not quite as good as those from Method 1 were obtained by Method 2 (Figure S1C), indicating that more than 4 terms were required in order to obtain a perfect fit. However, Method 2 was valuable in showing that without any operator guidance, exponential terms with $t_{1/2}$s spread over most of the recovery time course's data range were needed in order to even approach a reasonable fit, agreeing with conclusions deduced, from Table 1 and Figure 7, in the main paper.

---

[1] Synergy Software (2014) KaleidaGraph, Version 4.5.2 (Mac). www.synergy.com

**Table S1. Species (& acronyms therefor) for which individual data are given here & in main paper**

| species | acronym | botanical family | vernacular name | growth form[1] | native habitat[2] | branch source[3] |
|---|---|---|---|---|---|---|
| *Adenostoma fasciculatum* | Adefas | Rosaceae | chamise | S | CA L | CA L |
| *Arctostaphylos densiflora* | Arcden | Ericaceae | manzanita | S | CA L | CA L |
| *Arctostaphylos glandulosa* | Arcgla | Ericaceae | manzanita | S | CA L | CA L |
| *Betula nana* | Betnan | Betulaceae | dwarf birch | S | AK | AK |
| *Dendromecon rigidus* | Denrig | Papaveraceae | tree poppy | S | CA L | CA L |
| *Diospyros virginiana* | Diovir | Ebenaceae | persimmon | T | US E | CA L |
| *Gaultheria shallon* | Gausha | Ericaceae | salal | S | NWL | NWL |
| *Genista monspessulana* | Genmon | Fabaceae | French broom | LS/ST | MED | CA L[4] |
| *Liriodendron tulipifera* | Lirtul | Magnoliaceae | tulip tree | T | US E | CA L |
| *Metasequoia glyptostroboides*[5] | Metgly | Taxodiaceae | dawn redwood | T | CH | CA L |
| *Morus alba* | Moralb | Moraceae | mulberry | T | CH | CA L |
| *Populus tremuloides* | Poptre | Salicaceae | aspen | T | AK/MW | AK |
| *Pseudotsuga menziesii*[5] | Psemen | Pinaceae | Douglas fir | T | MW | CA L |
| *Salix glauca* | Salgla | Salicaceae | grayleaf willow | S | AK/MW | AK |
| *Salix pulchra* | Salpul | Salicaceae | tealeaf willow | S | AK | AK |
| *Umbellularia californica* | Umbcal | Lauraceae | California bay | T | CA L | CA L |

[1] S, shrub; T, tree; LS/ST, large shrub or small tree.

[2] Natural occurrence, much oversimplified: AK, interior Alaska; CA L, lowland (coastal) California; CH, China; MED, Mediterranean area; MW, American mountain west; NWL, coastal Oregon & Washington; US E, eastern U.S.

[3] Specimens from a different source than their native range were from horticultural plantings, except as noted below.

[4] Introduced, but thoroughly naturalized, in the far western U.S.

[5] Softwoods; the rest are hardwoods.

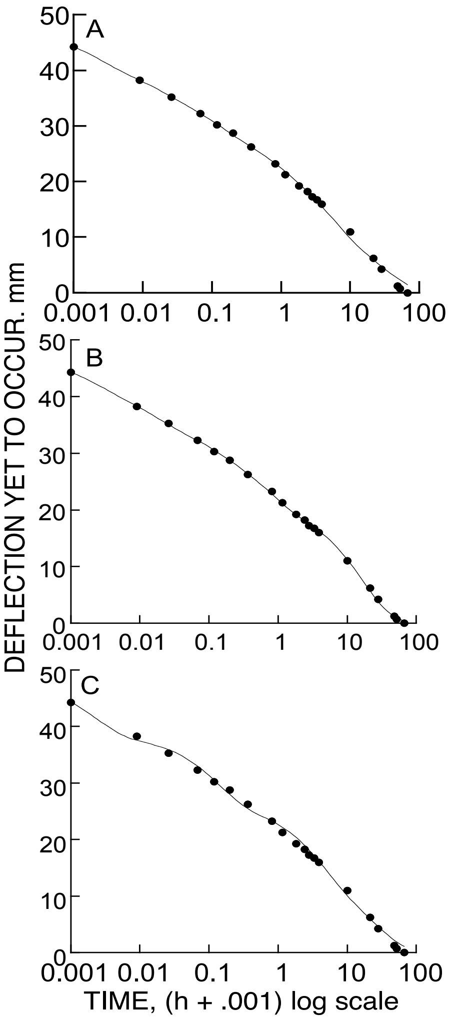


Figure S1. Results of computer curve-fitting, by a sum of exponential terms, of a post-unloading, retarded recovery time course of a branch segment from the shrub *Arctostaphylos glandulosa*. Marker points show the actual recovery data (same points in all 3 graphs); lines show the calculated fitting curves. (A), initially fitted by 6 terms whose coefficients $b_i$ in equation (2) were spread log-evenly (by factors of 7 fold) between 490 and 0.03 $h^{-1}$, the computer calculating values for the best-fitting coefficients $a_i$ [equation (2)]. (B), Improvement of fit in the right-hand part of profile (A) by moving to the right (decreasing) the $b_i$ values of the two smallest terms (largest $t_{1/2}$s) in (A) by factors of 7 and 3.5, respectively. (C), fitted by 4 terms for which the computer chose the values of both $a_i$ and $b_i$ in equation (2) for each of the terms. Sinuous departure of calculated curve from data points along the length of curve C indicates that >4 terms are needed for an exact fit.

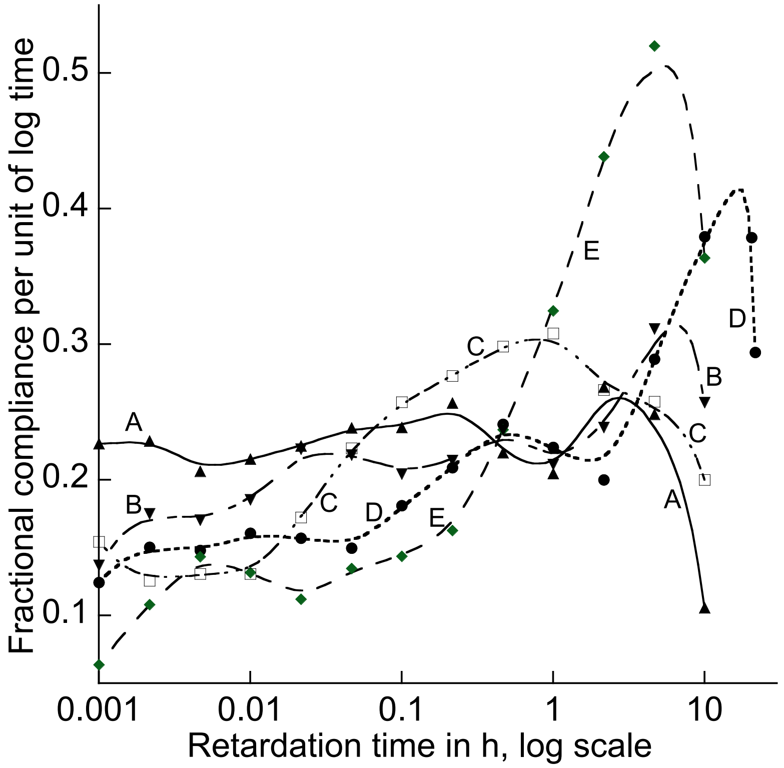


Figure S2. Calculated bending retardation spectra for woody branches from 5 species other than those of Figure 8 in the main paper. Each plot has a different line and marker point style, as well as a different letter identifying it which is shown in at least two places, so it is possible to follow each curve despite their intertanglement. These spectra were obtained in the same way as those of the main paper's Figure 8, but here show both the calculated points (the marker points) as well as the computer-drawn smooth curve through them, showing how the computer's smooth curve sometimes departs somewhat from extreme values of the markers. Species: (A) *Umbellularia californica,* (B) *Populus tremuloides*, (C) *Diospyros virginiana*, (D) *Arctostaphylos glandulosa*, (E) *Genista monspessulana*.
